# Creation of High-Density Néel Skyrmions by Interfacial-Proximity Engineering

**DOI:** 10.3390/ma19020340

**Published:** 2026-01-14

**Authors:** Tingjia Zhang, Chendi Yang, Xiaowei Lv, Ke Pei, Xiao Yang, Wuyang Tan, Junye Pan, Jiazhuan Qin, Meichen Wen, Wei Li, Jia Liang, Renchao Che

**Affiliations:** 1 School of Materials and Chemistry, University of Shanghai for Science and Technology, Shanghai 200093, China; 2 Laboratory of Advanced Materials, Shanghai Key Lab of Molecular Catalysis and Innovative Materials, State Key Laboratory of Coatings for Advanced Equipment, College of Smart Materials and Future Energy, Fudan University, Shanghai 200438, China; 3 Electron Microscopy Center, Institute of Science and Technology, Fudan University, Shanghai 200438, China; 4 College of Physics, Donghua University, Shanghai 201620, China; 5 Materials Genome Institute, Shanghai University, Shanghai 200444, China; 6 College of Smart Materials and Future Energy, State Key Laboratory of Photovoltaic Science and Technology, Fudan University, Shanghai 200433, China

**Keywords:** two-dimensional materials, van der Waals ferromagnets, magnetic skyrmion, van der Waals heterostructure, Dzyaloshinskii–Moriya interaction

## Abstract

Two-dimensional ferromagnets are promising for compact spintronic devices. However, their centrosymmetric structure inherently suppresses the Dzyaloshinskii–Moriya interaction (DMI), hindering the stabilization of chiral spin texture. Here, a tunable DMI induced by interface symmetry breaking in Fe_3_GeTe_2_/MoS_2_ vdW heterostructures is reported. We find that the interfacial DMI stabilizes Néel-type skyrmions in Fe_3_GeTe_2_/MoS_2_ heterostructures under zero magnetic field, with nucleation observed at 64 Oe and annihilation at 800 Oe via Lorentz transmission electron microscopy (LTEM). Skyrmion density peaks (~0.57 skyrmions/μm^2^) at a Fe_3_GeTe_2_ thickness of ~30 nm and decays beyond ~60 nm, indicating a finite penetration depth of the proximity effect. Such modulated DMI enables a stabilized nucleation of Néel type skyrmions, allowing for precise control over their density, revealed by Lorentz transmission electron microscopy. Thickness-dependent measurements confirm the interfacial origin of this stabilization. Skyrmion density reaches peak in thin Fe_3_GeTe_2_ layers and decays beyond ~60 nm, defining the finite penetration depth of the proximity effect. Micromagnetic simulations reproduce the field-dependent evolution of skyrmions, showing a strong correlation to interfacial DMI. First-principles calculations attribute this DMI to asymmetric charge redistribution and spin–orbit coupling at the heterointerface. This work establishes interface engineering as a universal strategy for stabilizing skyrmions in centrosymmetric vdW ferromagnets, offering a thickness-tunable platform for next-generation two-dimensional spintronic devices.

## 1. Introduction

The discovery of long-range ferromagnetic order in atomically thin van der Waals (vdW) crystals, exemplified by Cr_2_Ge_2_Te_6_, CrI_3_, and particularly Fe_3_GeTe_2_ with its relatively high Curie temperature. This finding has ignited intense research into ultra-compact, two-dimensional spintronic devices operating at the ultimate atomic-scale limit [1,2,3,4]. Realizing the full potential of these materials highly depends on a controllable nucleation of the chiral topological spin textures, such as skyrmions [5,6,7]. As topologically protected spin textures, skyrmions offer transformative potential for high-density spintronic devices by their nanoscale dimensions and ultralow current-driven mobility [8,9,10,11,12]. The stabilization of such non-trivial spin textures relies crucially on the Dzyaloshinskii–Moriya interaction (DMI), an antisymmetric exchange coupling that originates from broken inversion symmetry in conjunction with spin–orbit coupling (SOC) [13,14,15,16]. While the theoretical foundations of DMI were laid decades ago [17,18], its pivotal role in stabilizing chiral magnetic phases was spectacularly confirmed by the discovery of a skyrmion lattice in bulk MnSi [19], and later by the direct real-space observation of individual skyrmions using Lorentz transmission electron microscopy (LTEM) [20]. However, a significant challenge emerged when extending this paradigm to two-dimensional (2D) van der Waals ferromagnets [21,22]. Following the discovery of long-range ferromagnetism in few-layer Cr_2_Ge_2_Te_6_ and CrI_3_, the metallic ferromagnet Fe_3_GeTe_2_ emerged as a promising candidate due to its relatively higher Curie temperature and strong perpendicular magnetic anisotropy [23,24]. Yet, like most intrinsic 2D van der Waals magnets, its centrosymmetric crystal structure intrinsically suppresses DMI, hindering skyrmion formation. Consequently, artificially breaking the inversion symmetry has become an essential prerequisite. A key strategy is to construct van der Waals heterostructures. Early pioneering work demonstrated that coupling Fe_3_GeTe_2_ with non-centrosymmetric WTe_2_ could induce interfacial DMI and stabilize Néel-type skyrmions [25]. This established interface engineering as a potent and promising strategy, motivating further exploration of alternative heterostructure systems to achieve optimized and tunable skyrmion properties.

Interface engineering emerges as a powerful and universal strategy to overcome material-level symmetry limitations and to artificially tailor functionalities—a principle that has been successfully demonstrated across diverse fields, from the design of multimodal contrast agents in biomedical imaging to the customization of electronic and magnetic properties in condensed matter systems [26,27]. Building on this broad principle, interface engineering in artificially stacked vdW heterostructures offers a particularly potent route to break inversion symmetry and induce a tunable DMI at the atomic scale [28,29]. The key mechanism is the proximity coupling of adjacent layers composed of materials with strong spin–orbit coupling (SOC). When a vdW ferromagnetic layer contacts heavy elements in adjacent materials, the interface proximity effect disrupts local inversion symmetry and generates tunable interfacial DMI [30,31,32]. This proximity-enabled engineering has already achieved remarkable success beyond magnetism. Representative examples include proximitized spin–orbit effects and spin/valley polarization at the interfaces between graphene and transition metal disulfide compounds (e.g., WSe_2_, WS_2_), as well as the manipulation of spin or valley polarization in WSe_2_ coupled with magnetic CrI_3_ [33,34,35,36,37,38,39,40,41]. This proximity-induced spin and valley polarization offers a new degree of freedom for controlling site-specific nucleation and density of skyrmions in 2D magnets [42].

Achieving high–data-density storage generally requires materials that support small-sized and high-density skyrmions. To date, it has been demonstrated in heavy metal/ferromagnetic multilayer film, in which the combination of interface-driven perpendicular magnetic anisotropy (PMA) and DMI leads to the stabilization of Néel-type skyrmion [43,44,45]. In these systems, skyrmions nucleate randomly under an external magnetic field, creating large inter-skyrmion gaps and leading to relatively low areal densities. Therefore, achieving site-specific control over skyrmion formation and packing density is crucial for improving data storage capacity, yet it remains a significant challenge for practical skyrmion-based devices.

In this work, we demonstrated that the controllable nucleation of high-density Néel-type Skyrmions can be stabilized in high-quality Fe_3_GeTe_2_/MoS_2_ vdW heterostructures through interface-induced DMI. LTEM shows that, in contrast to pristine Fe_3_GeTe_2_, the heterostructure stablize skyrmions under zero-field conditions, a state unattainable in the standalone film under the same conditions. Furthermore, cooling the heterostructure under a smaller external magnetic field further increases the skyrmion density, and this phenomenon persists in subsequent magnetic field changes. It is worth noting that we have determined that this stability is dominated by the interface DMI. Across a broad range of thicknesses and applied fields, the heterostructure consistently exhibits higher skyrmion densities than pristine Fe_3_GeTe_2_. As the Fe_3_GeTe_2_ thickness increases, this effect gradually diminishes and approaches the bulk density, indicating a finite penetration depth of the proximity effect. Micromagnetic simulation, together with first-principles analysis of interfacial charge rearrangement and density correlation provide a mechanistic basis for inducing DMI. Our work established that Fe_3_GeTe_2_/MoS_2_ is an effective platform for stabilizing the topological spin texture in centrosymmetric vdW magnets through interface engineering, and defined the operating state by the layer thickness of tunable two-dimensional spintronic devices.

## 2. Materials and Methods

Fe_3_GeTe_2_ single crystals were grown via the chemical vapor transport (CVT) method using stoichiometric mixtures of Fe (99.99%), Ge (99.99%), and Te (99.999%) powders with iodine as the transport agent. The sealed quartz tube was heated to a temperature gradient of 780–700 °C for 7 days and cooled naturally.

For heterostructure fabrication, a few layers of MoS_2_ were dry-transferred onto Fe_3_GeTe_2_ flakes using a commercial two-dimensional material transfer platform (META, Nanjing, China) under an optical microscope. The transfer was performed at a stage temperature of 70 °C to reduce polymer residue. A contact pressure of approximately 0.7 MPa was applied for 2 minutes to ensure intimate van der Waals contact, followed by a slow release of the stamp. The heterojunction was assembled on a silicon nitride (SiN) substrate to ensure compatibility with subsequent transmission electron microscopy (TEM) characterization. Atomic force microscopy (AFM, Bruker Dimension Icon, Billerica, MA, USA) confirmed interfacial flatness and layer thickness uniformity.

Magnetic domain imaging was performed using a Lorentz transmission electron microscope (JEM-2100F, JEOL Ltd., Tokyo, Japan) at 200 kV. Experiments were conducted at a constant temperature of 110 K using a liquid-nitrogen cooling holder. An in-plane magnetic field was applied using a dedicated sample holder (Gatan, Pleasanton, CA, USA) with a calibrated magnetic field range of 0 to 1 T. The field was swept at a rate of 50 Oe/s during measurements. The sample was tilted to 11° relative to the electron beam to maximize magnetic phase contrast. The objective lens was closed during sample insertion to maintain the magnetic structure. Two distinct cooling protocols were employed: (i) Zero-field cooling (ZFC): the sample was cooled from 300 K to 110 K with no applied magnetic field. (ii) Field cooling (FC): the sample was cooled from 300 K to 110 K under a constant in-plane magnetic field of 250 Oe. Raman spectroscopy (Renishaw inVia, 532 nm laser, New Mills, UK) was employed to verify the chemical composition of pristine Fe_3_GeTe_2_ and Fe_3_GeTe_2_/MoS_2_ heterostructures by identifying characteristic vibrational modes of Fe-Ge-Te bonding (~120 cm^−1^) and MoS_2_-derived phonon signatures (~380 cm^−1^), while simultaneously probing interfacial strain through spectral shifts. Micromagnetic simulations based on the LLG function were performed by using the MuMax3 software (2.1.2) to reproduce the multiple transitions. Slab geometries of dimensions 400 nm × 400 nm × 20 nm were used, with a rectangle mesh of size 2 nm × 2 nm × 2 nm. The uniaxial anisotropy constant (Kμ) and exchange stiffness constant (A1) were chosen based on the experimentally established values. The Gilbert damping stiffness constant ɑ was set to 0.3. To show the structure particularity of 2D materials, the exchange stiffness constant between layers (A2) was set to 1 × 10^−13^ J m^−1^. All equilibrium states were obtained by fully relaxing randomly distributed magnetization.

The Fe_3_GeTe_2_/MoS_2_ interface was investigated using density functional theory (DFT). A coherent supercell was constructed to model the heterostructure, containing a unit cell of Fe_3_GeTe_2_ stacked atop a commensurate unit cell of monolayer MoS_2_, with a vacuum layer of >15 Å to avoid inter-slab interactions. All the calculations are performed in the generalized gradient approximation proposed by Perdew, Burke, and Ernzerhof, which is selected for the exchange-correlation potential. The plane-wave energy cutoff and k-point mesh were determined through convergence tests (see Supplementary Figure S1 for details), leading to the selection of a cutoff energy of 400 eV and a 3 × 3 × 3 k-point for Brillouin zone integration. All the structures are relaxed until the residual forces on the atoms have declined to less than 0.01 eV/Å.

## 3. Results and Discussion

### 3.1. Interface Engineering and Structural Verification

**Figure 1:**
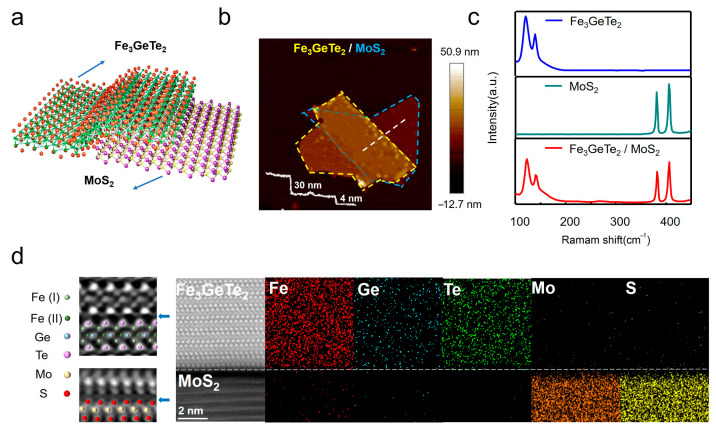
**Structural characterization of Fe_3_GeTe_2_/MoS_2_ heterostructures.** (**a**) Schematic of the van der Waals heterostructure with Fe_3_GeTe_2_ stacked atop MoS_2_. (**b**) AFM topography showing Fe_3_GeTe_2_ (yellow dashed outline) on MoS_2_ (blue dashed outline); height profile (white line) confirms Fe_3_GeTe_2_ (~30 nm) and MoS_2_ (~4 nm) thicknesses. Scale bar: 2 μm. (**c**) Raman spectra of pristine Fe_3_GeTe_2_, MoS_2_, and the heterostructure. (**d**) Cross-sectional STEM image (**left**) with atomic resolution at the Fe_3_GeTe_2_-MoS_2_ interface. EELS maps (**right**) confirm elemental segregation (Fe/Ge/Te in Fe_3_GeTe_2_; Mo/S in MoS_2_). Scale bar: 5 nm.

To establish a structural foundation for exploring interfacial driven topological spin textures, we first prepared Fe_3_GeTe_2_/MoS_2_ heterostructures by dry transfer technology and characterized their morphology and structures. Figure 1a presents the schematic diagram of the vdW heterostructure, in which a Fe_3_GeTe_2_ is vertically stacked on a few-layer MoS_2_ substrate. The atomically smooth interface ensures tight vdW coupling without the formation of chemical bonds. Atomic force microscopy (AFM) image in Figure 1b clearly shows the surface morphology, where the MoS_2_ and Fe_3_GeTe_2_ regions appear as distinct terraces (marked with yellow and blue dashed lines). The height profile extracted from the AFM image confirm the MoS_2_ thickness of ~4 nm and a Fe_3_GeTe_2_ overlayer of ~30 nm. Additional heterostructures with Fe_3_GeTe_2_ thicknesses in the 30–60 nm range were prepared to investigate thickness-dependent interfacial effects.

Raman spectroscopy in Figure 1c further verifies the chemical integrity of the constituent layers. Pristine Fe_3_GeTe_2_ shows a characteristic vibrational mode at ~120 cm^−1^, which belongs to the vibration of the Fe-Ge-Te bond, whereas the MoS_2_ layer shows its signature phonon modes at ~380 cm^−1^ (E_2g_^1^) and ~408 cm^−1^ (A_1g_) [46,47]. Importantly, the heterostructure spectrum retains all characteristic peaks of both materials without obvious displacement, confirming the preservation of individual material properties and negligible interfacial strain or chemical reactions.

To directly observe the interfacial structure at the atomic scale, cross-sectional spherical aberration-corrected scanning transmission electron microscopy (STEM) and electron energy-loss spectroscopy (EELS) were carried out. As shown in Figure 1d, the high-resolution STEM image (left) shows a clear and atomically defined interface, with no detectable interdiffusion between Fe_3_GeTe_2_ and MoS_2_. Elemental mapping via EELS confirms clear spatial segregation: Fe, Ge and Te are confined to the Fe_3_GeTe_2_ layer, whereas Mo and S are exclusively localized in the MoS_2_ layer. These atomic-scale characterizations jointly validate the successful construction of high-quality, van der Waals interfaces with pronounced symmetry breaking. The atomically sharp boundary and absence of interdiffusion demonstrate a high degree of interfacial perfection, which is crucial for realizing a well-defined and reproducible proximity-induced DMI—the foundational mechanism enabling the stabilization of topological spin textures in this heterostructure system.

### 3.2. Skyrmion Dynamic Response

Prior to probing the topological spin textures, we verified the intrinsic magnetic properties of the Fe_3_GeTe_2_ constituent. Magnetization versus temperature (M-T) measurements (Supplementary Figure S2) confirm a Curie temperature (T_C_) of ~220 K. This establishes that our systematic Lorentz TEM investigations, conducted at 110 K, are performed within a stable ferromagnetic phase well below the magnetic ordering transition. At this measurement temperature, field-dependent magnetization (M-H) curves (Supplementary Figure S3) exhibit square hysteresis loops with high remanence when the magnetic field is applied perpendicular to the sample plane, unambiguously demonstrating the strong perpendicular magnetic anisotropy essential for the formation and observation of Néel-type skyrmions.

**Figure 2:**
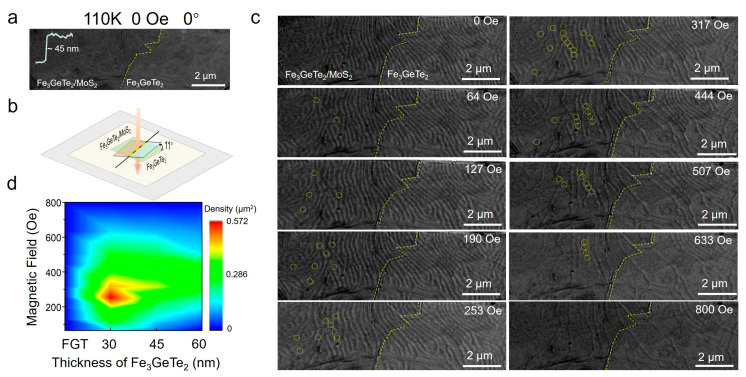
**Néel skyrmions formation and dynamics in 45 nm Fe_3_GeTe_2_/4 nm MoS_2_ heterostructures under zero-field cooling.** (**a**) Lorentz TEM image at 0° tilt (0 Oe) showing no magnetic contrast, confirming out-of-plane magnetization of Néel skyrmions. Scale bar: 2 μm. (**b**) Lorentz TEM images of magnetic domains at 11° tilt under varying magnetic fields (0–800 Oe). Yellow circles mark Néel-type skyrmions. Scale bar: 2 μm. (**c**) Schematic of the experimental setup with 11° sample tilt relative to the electron beam. (**d**) Magnetic field and thickness dependence of skyrmions density for pure Fe_3_GeTe_2_, Fe_3_GeTe_2_/MoS_2_ heterostructures with different thickness of Fe_3_GeTe_2_ (30 nm, 45 nm and 60 nm) and the thickness of MoS_2_ is 4 nm. The yellow dashed lines indicate the boundaries between the heterostructure and the pure phase regions.

To investigate the topological spin texture induced by interfacial DMI, we conducted AFM on the Fe_3_GeTe_2_/MoS_2_ heterostructure with Fe_3_GeTe_2_ and MoS_2_ thicknesses of 45 nm and 4 nm, respectively (Figure 2). With this material foundation established, we conducted Lorentz TEM measurements. The heterostructure was fabricated on a flat, inert silicon nitride (SiN) substrate, which served solely as a non-magnetic support film for TEM compatibility and introduced negligible strain or magnetic interference (Supplementary Figure S4). Under these conditions, no magnetic contrast was observed at 0° tilt (Figure 2a), whereas clear contrast emerged when the sample was tilted to 11° (Figure 2b)—a behavior that is characteristic of out-of-plane magnetization. To definitively link our experimental observation (tilt-dependent contrast) to the material property (PMA) and the specific spin texture (Néel skyrmion), we performed micromagnetic simulations of the LTEM contrast [48,49]. The simulations (Supplementary Figure S5) confirm that for a Néel-type skyrmion with out-of-plane magnetization, observable phase contrast requires a tilted geometry (e.g., ±11°), while it remains negligible at 0° tilt, in perfect agreement with our experiments. This consolidated evidence conclusively attributes the observed contrast behavior to the out-of-plane magnetization of the stabilized Néel skyrmions.

Figure 2c presents a series of Lorentz TEM images taken at an 11° sample tilt under magnetic fields varying from 0 to 800 Oe. Yellow circles mark distinct Néel-type skyrmions, which nucleate at 64 Oe, increase in density with increasing the magnetic field, reach a maximum density at 317 Oe, and ultimately vanish by 800 Oe. The 11° tilt is essential for visualizing these topological structures: it aligns the electron beam with the out-of-plane magnetization components of the Néel skyrmions, generating Lorentz force-induced phase contrast that reveals their characteristic (vortex) morphology. This geometry guarantees that the subtle magnetic structure of the skyrmions is resolved. Moreover, the absence of contrast at 0° sample tilt (Figure 2a) further confirms that the spin textures possess predominantly out-of-plane magnetization. Specifically, when the electron beam is parallel to the magnetic moments, phase shifts induced by the Lorentz force are nullified. This behavior represents a crucial signature of Néel-type skyrmions that are stabilized by interfacial symmetry breaking and DMI.

Moreover, Lorentz TEM measurements were also carried on the Fe_3_GeTe_2_/MoS_2_ heterostructure with different Fe_3_GeTe_2_ thicknesses (30 nm and 60 nm), as shown in Supplementary Figures S6 and S7. Quantitative analysis of skyrmion density (Figure 2d) reveals a strong dependence on both magnetic field and Fe_3_GeTe_2_ thickness. A 2D color map shows that all heterostructures exhibit maximum skyrmion density at intermediate magnetic fields (190–253 Oe), with thinner Fe_3_GeTe_2_ layers supporting significantly higher densities. For example, the Fe_3_GeTe_2_/MoS_2_ heterostructure with the Fe_3_GeTe_2_ thickness of 30 nm achieves a peak density of ~0.57 skyrmions/μm^2^ at 253 Oe, whereas the 45 nm and 60 nm counterparts yield lower peaks of ~0.29 and ~0.14 skyrmions/μm^2^, respectively. This thickness dependence directly correlates with the interfacial volume ratio: thinner Fe_3_GeTe_2_ layers experience a stronger proximity-induced DMI from the MoS_2_ interface, stabilizing more skyrmions. The MoS_2_ layer, with its strong spin–orbit coupling and structurally sharp interface, is thus the key element that breaks the inversion symmetry and introduces the interfacial DMI, enabling skyrmion stabilization. In contrast, pure 60 nm Fe_3_GeTe_2_ (Supplementary Figure S7) shows negligible skyrmion formation (<0.05 skyrmions/μm^2^), confirming that interfacial DMI is essential for stabilizing these topological spin textures. The critical nucleation and annihilation fields were determined by identifying the magnetic field values at which skyrmions first appeared and completely disappeared, respectively, during field-cycling LTEM measurements at 110 K. To confirm reproducibility across multiple regions and samples, we performed repeated measurements on three independently fabricated Fe_3_GeTe_2_/MoS_2_ heterostructures for each thickness (30, 45, and 60 nm). Representative Lorentz TEM images from different regions and applied magnetic fields, illustrating the determination of these critical events, are compiled in Supplementary Figure S8. Statistical analysis of the data yielded nucleation fields in the range of 60–130 Oe and annihilation fields of 650–800 Oe, with the detailed distribution presented in Supplementary Figure S9.

The density map further reveals that the magnetic field window for stable skyrmions existence narrows with increasing Fe_3_GeTe_2_ thickness, from 64 to 507 Oe for 30 nm Fe_3_GeTe_2_ to 127–317 Oe for 60 nm Fe_3_GeTe_2_, indicating that thicker films are more susceptible to field-induced annihilation due to diluted interfacial effects. The interfacial DMI-driven stabilization mechanism is fundamentally robust across a wide temperature range within the ferromagnetic phase. The DMI originates from the broken inversion symmetry and proximity-induced spin–orbit coupling at the heterointerface—effects that are intrinsic to the interface and not confined to 110 K. Therefore, the ability to stabilize Néel-type skyrmions via this interface-engineering strategy is expected to persist throughout the ferromagnetic phase, up to the Curie temperature (*T*_C_ ≈ 220 K). Quantitative aspects of the stability window (e.g., nucleation and annihilation fields) will naturally vary with temperature due to the thermal dependence of magnetic parameters such as saturation magnetization (*M_S_*) and anisotropy, but the essential DMI stabilization mechanism remains active. Collectively, these results demonstrate that the Fe_3_GeTe_2_/MoS_2_ interface introduces significant interfacial DMI, stabilizing Néel-type skyrmions absent in pristine Fe_3_GeTe_2_ [25]. The thickness tunability of skyrmion density—enhanced in thinner Fe_3_GeTe_2_ layers and suppressed in thicker films—provides a robust pathway for engineering skyrmion-based van der Waals (vdW) heterostructures, a key requirement for next-generation spintronic devices requiring precise control over topological spin textures.

**Figure 3:**
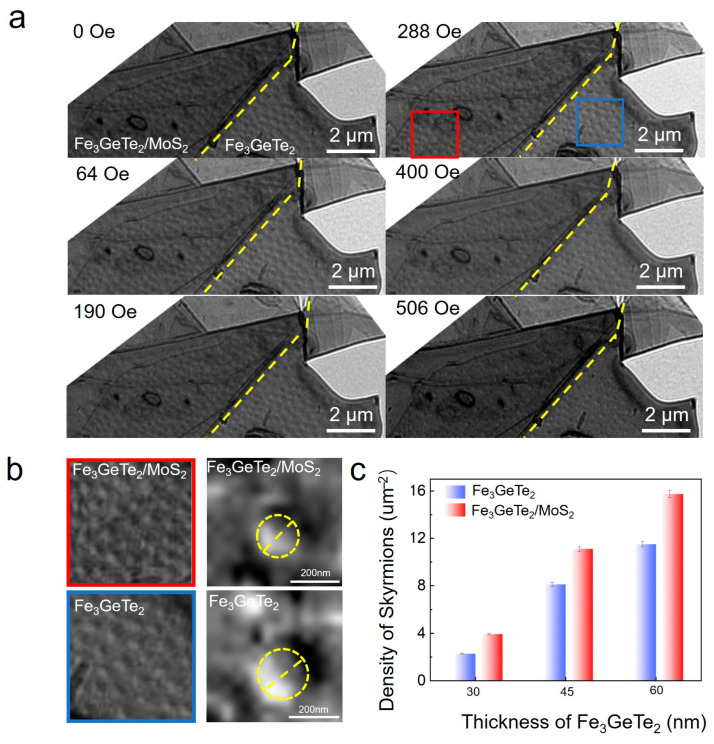
**Magnetic domain evolution and skyrmion density characteristics in Fe_3_GeTe_2_/MoS_2_ heterostructures.** (**a**) Lorentz TEM images showing magnetic domain evolution in Fe_3_GeTe_2_/MoS_2_ heterostructures (left region) and pure Fe_3_GeTe_2_ (right region, marked by dashed yellow line) under varying magnetic fields (0–506 Oe). Scale bar: 2 μm. (**b**) Comparative high-magnification Lorentz transmission electron microscopy (TEM) images of Néel-type skyrmions in the same unit area of pristine Fe_3_GeTe_2_ (blue frame) and 30 nm Fe_3_GeTe_2_/MoS_2_ heterostructure (red frame) under an applied magnetic field of 228 Oe, and corresponding magnified views of individual skyrmions (bottom row) to highlight their morphological details. Scale bar: 200 nm. (**c**) Skyrmion density as a function of Fe_3_GeTe_2_ thickness for pure Fe_3_GeTe_2_ and Fe_3_GeTe_2_/MoS_2_ heterostructures. Error bars represent the standard deviation derived from three independently fabricated heterostructures, confirming the reproducibility of the skyrmion stabilization effect.

To further examine the influence of field cooling (FC) on skyrmion stability, we conducted Lorentz TEM measurements on the Fe_3_GeTe_2_/MoS_2_ heterostructure with the Fe_3_GeTe_2_ thickness of 30 nm cooled to 110 K under an applied magnetic field of 190 Oe (Figure 3). Unlike the case of zero-field cooling (ZFC), where skyrmions nucleate only above 64 Oe, discrete Néel-type skyrmions are clearly visible even at 0 Oe under FC conditions (Figure 3a), indicating that the applied cooling field stabilizes residual topological textures. When the magnetic field increases to 190 Oe, the skyrmion density rises sharply, forming a hexagonal skyrmion lattice before they gradually annihilate at higher magnetic fields (>500 Oe). This extension of skyrmion stability to zero field, along with enhanced nucleation in the intermediate field regime, indicates that FC preconditions the heterostructure for skyrmion formation, which is a key advantage for device applications requiring non-volatile spin textures.

High-magnification images at 228 Oe (Figure 3b) compare the magnetic response of the Fe_3_GeTe_2_/MoS_2_ heterostructure with pristine Fe_3_GeTe_2_. Both exhibit circular skyrmions with clear contrast, and the observed light-dark contrast at the edges confirms their Néel-type morphology. Notably, skyrmions in pristine Fe_3_GeTe_2_ have an approximate diameter of ~200 nm, whereas those in the heterostructure are smaller (~170 nm). This comparison directly verifies the influence of the MoS_2_ interface on skyrmion size.

In addition to the Fe_3_GeTe_2_/MoS_2_ heterostructure with the Fe_3_GeTe_2_ thickness of 30 nm, the same measurements were also performed on Fe_3_GeTe_2_/MoS_2_ heterostructures with Fe_3_GeTe_2_ thicknesses of 45 nm and 60 nm, as shown in Supplementary Figures S10 and S11, respectively. Quantitative analysis of skyrmion density across all samples, including and pristine Fe_3_GeTe_2_ and heterostructures with variable Fe_3_GeTe_2_ thicknesses (30, 45, 60 nm) (Figure 3c), further reinforces the dominant role of interfacial effects. For heterostructures, the skyrmion density in the heterostructure region is consistently higher than that in the pristine Fe_3_GeTe_2_ region regardless of thickness, indicating that interfacial proximity effectively enhances skyrmion formation. Remarkably, the heterostructure with the Fe_3_GeTe_2_ thickness of 60 nm exhibits the highest skyrmion density, underscoring that even at this thickness—approaching the previously determined critical penetration depth of the proximity effect—the heterostructure still maintains a density superior to that of pristine Fe_3_GeTe_2_, highlighting the robustness of interfacial DMI. The small error bars underscore the high consistency across multiple samples, which is attributable to the highly consistent interfaces achieved by our dry-transfer process, as corroborated by AFM and STEM-EELS (Figure 1b,d). Our multi-scale characterization confirms that these interfaces are atomically sharp and free from significant interdiffusion. Establishing this consistent interfacial baseline was essential for our study, as it allows the observed topological magnetism to be clearly attributed to the proximity-induced DMI rather than to sample-to-sample variability. This consistent interfacial quality was essential for attributing the observed topological magnetism to the proximity-induced DMI.

These FC results complement our ZFC studies, showing that an external field during cooling can pre-nucleate skyrmions and extend their stability to lower fields. When combined with the thickness-dependent density enhancement, our findings establish that interfacial DMI is not only a necessary condition for skyrmion formation in centrosymmetric vdW magnets but also tunable via layer thickness. This dual control of nucleation via field cooling and density optimization via thickness engineering provides a practical approach for designing controllable topological spin textures.

The observation that the proximity-induced interfacial DMI significantly modulates the magnetic state of Fe_3_GeTe_2_ layers up to ~60 nm thick, as evidenced by the decay of skyrmion density (Figure 2d and Figure 3c), is a key finding. This effective range, extending well beyond the atomic interface, is physically consistent with the mechanism of exchange-mediated propagation. The chiral DMI originates at the interface but is transmitted into the ferromagnetic bulk via the strong exchange coupling, which coherently aligns neighboring spins. Consequently, the interfacial effect can perturb the spin texture over tens of nanometers, rather than being confined to a few atomic layers. This behavior is analogous to the well-documented long-range nature of interfacial DMI in conventional heavy metal/ferromagnet thin-film multilayers, where the DMI effectively influences magnetic properties throughout ferromagnetic layers of comparable thickness [50,51,52]. The ~60 nm scale thus represents the depth over which the exchange-propagated interfacial DMI competes with and overcomes the bulk magnetic energies favoring collinear order, defining a critical design parameter for proximity-engineered van der Waals spintronics. This interpretation is quantitatively supported by the micromagnetic simulations presented in the following section.

The choice of MoS_2_ with a thickness of ~4 nm in this model system is based on a balanced consideration for a clear proof-of-principle demonstration. This thickness provides optimal mechanical stability for reliable transfer and ensures the formation of an atomically sharp interface, which is crucial for inducing a well-defined interfacial effect. We note that the proximity-induced DMI is an interface-localized phenomenon; thus, beyond a few layers necessary for robust electronic properties, additional thickness of the SOC-providing layer (MoS_2_) does not significantly enhance the interaction but may increase the distance and technical complexity. To further validate the role of MoS_2_ thickness, we also fabricated a heterostructure with a thicker MoS_2_ flake (~8 nm) coupled with 30 nm Fe_3_GeTe_2_. Zero-field-cooled Lorentz TEM imaging (Supplementary Figure S12) confirmed that Néel-type skyrmions could still be stabilized, demonstrating the robustness of the interfacial proximity effect. However, the skyrmion density was lower than that in the ~4 nm MoS_2_ heterostructure, indicating that beyond an optimal thickness, additional MoS_2_ layers do not significantly enhance the DMI strength but may introduce increased distance and possible decoherence effects. This observation further supports our selection of ~4 nm MoS_2_ as an optimal balance between strong SOC, structural stability, and minimal thickness for practical device integration.

Beyond optimizing the thickness, the selection of MoS_2_ itself—over other transition metal dichalcogenides (TMDs) such as WSe_2_ or WS_2_ which possess stronger intrinsic spin–orbit coupling—was deliberate for this foundational study. MoS_2_ offers superior experimental accessibility and exceptional environmental stability, which are paramount for achieving the high-quality, reproducible interfaces required to unambiguously establish the causal link between interfacial symmetry breaking and DMI. Its moderate SOC strength is sufficient to induce a measurable DMI effect while providing a cleaner platform to isolate the role of interfacial symmetry breaking, minimizing potential complexities associated with extremely strong SOC.

While this study focuses on the Fe_3_GeTe_2_/MoS_2_ heterostructure as a prototypical system, the interfacial-proximity engineering strategy demonstrated here is underpinned by a general physical principle. The essential requirement is symmetry breaking coupled with proximity-induced spin–orbit effects from an adjacent material, a mechanism not unique to the MoS_2_ interface. This is directly evidenced by prior work showing stabilized Néel skyrmions in a related Fe_3_GeTe_2_/WTe_2_ heterostructure [25]. More broadly, the capability of various strong-SOC TMDs (e.g., WSe_2_, WS_2_) to impart significant spin–orbit coupling and spin-momentum locking to neighboring layers via proximity has been well established in graphene/TMD heterostructures [35,37]. These studies strongly suggest that substituting MoS_2_ with other TMDs would similarly induce interfacial DMI, potentially with tunable strength depending on the specific SOC Hamiltonian. We therefore anticipate that the strategy presented here can be extended to a wide range of two-dimensional magnets coupled with diverse high-SOC materials, opening a rich parameter space for tailoring DMI strength and skyrmion properties (such as size, stability, and nucleation field). Systematic future studies exploring these dimensions will be crucial for mapping the design rules of skyrmion-based van der Waals spintronic devices.

The LTEM observations and interfacial DMI analysis in this work were conducted at 110 K, a temperature aligned with the robust ferromagnetic regime of ultrathin Fe_3_GeTe_2_, whose Curie temperature is inherently limited. It is important to recognize that the thermal stability of skyrmions in this model system is fundamentally capped by the magnetic ordering temperature of the host ferromagnetic layer. Our study is therefore a proof-of-principle demonstration, with its primary achievement being the establishment of a universal interfacial-proximity strategy to induce substantial DMI and stabilize Néel skyrmions in otherwise centrosymmetric van der Waals magnets. The key parameters quantified here—such as the critical DMI strength and its finite penetration depth (~60 nm)—are universal design rules. These insights provide a direct blueprint for future efforts aimed at room-temperature operation: the identified mechanism can be directly transferred to van der Waals magnets with higher intrinsic ordering temperatures. Promising strategies to achieve this include ion intercalation or gating (which can dramatically enhance *T*_C_ in similar 2D magnets), compositional alloying and the use of emerging high-*T*c van der Waals ferromagnets [24,53,54]. Meanwhile, the elucidated roles of interface quality and SOC strength offer clear optimization targets. Thus, this work lays the essential foundational physics and material guidelines for advancing toward practical skyrmionics.

### 3.3. DMI-Driven Skyrmion Mechanism

To quantitatively correlate the emergence of Néel-type skyrmions with the interfacial DMI in the Fe_3_GeTe_2_/MoS_2_ heterostructure, we determined the DMI strength from the magnetic domain width observed by Lorentz TEM. As shown in Supplementary Figure S13, the heterostructure presents well-arranged fringe domains. The widths of these domains were measured at three positions and their average values were calculated to be approximately *W*_0_ = 200 ± 10 nm. This domain width is directly related to the domain wall energy, δ_W_, through the relation [25]:

(1) $$\delta_{W}^{with\;DMI}=\beta \frac{4\pi \delta_{W}}{M_{S}^{2}}$$

where ***Ms*** is the saturation magnetization and ***β*** is a material-specific parameter. For the heterostructure, we derived a domain wall energy of $\delta_{W}^{with\;DMI}$ = 0.53 mJ/m^2^.

For pristine Fe_3_GeTe_2_, where DMI is negligible, the domain wall energy can be simplified as $\delta_{W}^{no\;DMI}=4\sqrt{AK_{eff}}$ yielding a reported value of 3.9 mJ/m^2^ [25]. The exchange stiffness (***A***) and effective anisotropy constant (***K_eff_***) were adopted from established literature values for Fe_3_GeTe_2_ [25,55,56], which provides a reliable basis for comparative analysis. The DMI strength ***|D|*** was then calculated using the relation [25]:

(2) $$\delta_{W}^{with\;DMI}=\delta_{W}^{no\;DMI}-\pi |D|$$

The resulting DMI strength for the heterostructure is ***|D|*** = 1.07 ± 0.05 mJ/m^2^. This value substantially exceeds the critical threshold for Néel skyrmion stability, ***D_C_*** ≈ 0.1 mJ/m^2^, confirming the interfacial DMI is strong enough to stabilize chiral textures. The exchange stiffness ***A*** and effective anisotropy constant ***K_eff_*** used in our analysis are well-established literature values for Fe_3_GeTe_2_ [25,55,56]. For the core physics addressed here—the relative change in DMI induced by the interface and the resulting evolution of magnetic textures—using these accepted intrinsic parameters as a benchmark is a reasonable and common practice. The validity of this approach is directly corroborated by the consistency between its outcome and our multi-faceted experimental observations: the DMI strength derived (~1.07 mJ/m^2^) is not only comparable to values reported in analogous systems (e.g., WTe_2_/Fe_3_GeTe_2_), but more importantly, its predicted consequences (i.e., zero-field stability of Néel skyrmions, non-monotonic thickness-dependent density modulation) are in excellent agreement with our direct LTEM observations (Figure 2 and Figure 3). This self-consistency validates the applicability of the analytical framework to our system.

Given the strength of this DMI, a critical subsequent question is whether it dominates over magnetostatic interactions in our thickness range (30–60 nm). The theoretical criterion is clear: the measured|D|far exceeds Dc, sufficient to enforce the Néel chirality against the magnetostatically favored Bloch-type configurations. Experimentally, two key observations provide decisive evidence: (1) Skyrmion formation is negligible in pristine Fe_3_GeTe_2_ (Figure 2d), which shares an identical magnetostatic energy landscape; (2) The skyrmion density in the heterostructure exhibits a non-monotonic dependence on Fe_3_GeTe_2_ thickness, peaking at ~30 nm (Figure 2d and Figure 3c)—a hallmark of an interface-localized effect rather than a bulk magnetostatic one. Therefore, the interfacial DMI is unambiguously the dominant stabilization mechanism.

**Figure 4:**
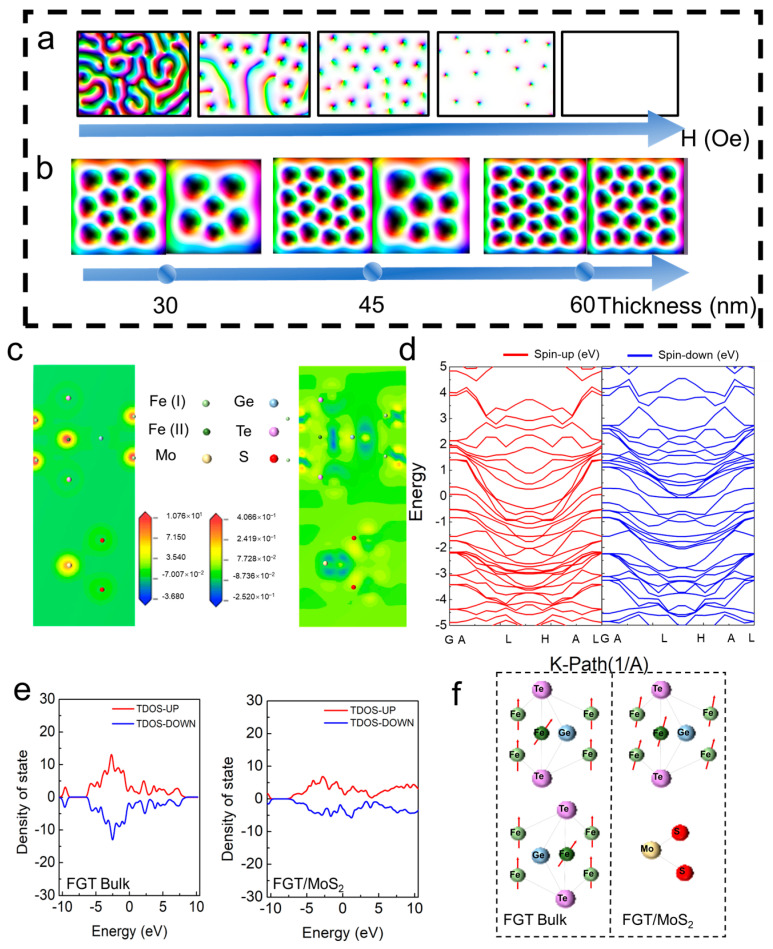
**Micromagnetic simulation of magnetic structure and interface electronic structure of Fe_3_GeTe_2_/MoS_2_ heterostructure.** (**a**) Micromagnetic simulation of the magnetic domain evolution of Fe_3_GeTe_2_/MoS_2_ heterostructures under different magnetic fields. (**b**) Micromagnetic simulation of Skyrmion lattice patterns of Fe_3_GeTe_2_/MoS_2_ heterostructures (**left**) with different thicknesses (30/45/60 nm) and the corresponding Fe_3_GeTe_2_ (**right**). (**c**) Charge density distribution at the Fe_3_GeTe_2_/MoS_2_ interface. (**d**) The band structure of the heterostructure near the Fermi level. (**e**) Spin polarization density of states (DOS) of pure Fe_3_GeTe_2_ and heterostructures. (**f**) Schematic diagram of interface spin coupling and symmetry breaking.

Having established the dominance of the interfacial DMI, we proceed to elucidate its microscopic origin and the physical basis of its thickness dependence. To this end, we combined micromagnetic simulation (MuMax3, version 2.1.2) with first-principles calculations [57]. This multiscale approach links experimentally observed magnetic textures to the behavior of electrons and magnetism at the atomic scale. Micromagnetic simulations were conducted using MuMax3 (2.1.2), assuming a moderate interfacial DMI strength (D = 1.0 mJ/m^2^) for Fe_3_GeTe_2_/MoS_2_ heterostructure and Fe_3_GeTe_2_ films of varying thicknesses (30–60 nm). The simulations successfully reproduce the experimental evolution of skyrmion nucleation and kinetics (Figure 4a,b). Figure 4a presents a micromagnetic simulation of the magnetic domain evolution of a Fe_3_GeTe_2_/MoS_2_ heterostructure varying with an applied magnetic field under the ZFC condition. Starting from the striped domain structure consistent with the initial magnetic state of the bulk Fe_3_GeTe_2_ in the low field, the system transitions to the Néel-type skyrmion lattice in the intermediate field and then saturates to uniform out-of-plane magnetization in the high field.

To explore thickness-dependent skyrmion stability under FC condition, we simulated the skyrmion densities in Fe_3_GeTe_2_/MoS_2_ heterostructures (left) and pristine Fe_3_GeTe_2_ (right) with different Fe_3_GeTe_2_ thicknesses (30, 45, and 60 nm) under 190 Oe (Figure 4b). The simulated results directly corroborate the experimental trends: the heterostructures exhibit significantly higher skyrmions densities than pristine Fe_3_GeTe_2_ at all thicknesses. These results verify that interfacial DMI plays a dominant role in the stability of skyrmions. As Fe_3_GeTe_2_ thickness increases beyond the critical penetration depth of the proximity effect, the DMI contribution progressively weakens, consistent with the experimentally observed convergence toward block-like magnetic textures.

First-principles calculations reveal that the interfacial DMI originates from asymmetric charge redistribution at the Fe_3_GeTe_2_/MoS_2_ interface, which generates a local electric field that couples with spin–orbit interactions. By correlating the interfacial electronic structure with micromagnetic dynamics, we establish a universal framework for engineering topological spin textures in centrosymmetric vdW magnets, enabling thickness-tunable skyrmion platforms for ultralow-power racetrack memories and logic devices. Figure 4c shows the calculated charge density and charge density difference (Δρ = ρ_hetero_ − ρ_Fe3GeTe2_ − ρ_MoS2_) of the Fe_3_GeTe_2_/MoS_2_ heterostructure, as well as the charge density of pristine Fe_3_GeTe_2_ (Supplementary Figure S14).

In pristine Fe_3_GeTe_2_, the charge density and differential charge density is uniformly distributed across the Fe-Ge-Te layer, consistent with its centrosymmetric crystal structure. In contrast, the Fe_3_GeTe_2_/MoS_2_ heterostructures exhibit obvious charge rearrangement at the interface: electron accumulation (yellow) occurs in the MoS_2_ layer, accompanied by charge depletion (blue) in the adjacent Fe_3_GeTe_2_ layer. The differential charge density is predominantly localized on the interfacial Fe and Te atoms, while the change around Ge atoms is comparatively minor. This indicates that the proximity-induced SOC is mediated primarily through the interaction between the MoS_2_ layer and the Fe/Te atomic planes at the interface. The dominant role of Fe and Te atoms can be rationalized by their stronger spin–orbit coupling and more active frontier orbitals involved in interfacial bonding compared to Ge. Thus, the DMI originates from a collective interfacial effect where symmetry breaking enables SOC from MoS_2_ to selectively couple with specific atomic sites (Fe, Te) in Fe_3_GeTe_2_, generating the chiral exchange interaction.

Band structure analysis of the heterostructure further confirms these symmetry-breaking effects, consistent with the emergence of DMI-driven skyrmions observed experimentally. Band structure analysis indicates that pristine Fe_3_GeTe_2_ exhibits symmetrical band dispersion around the Γ point (Supplementary Figure S15), which is a hallmark of its centrosymmetric crystal structure. In contrast, the heterostructure shows pronounced band asymmetry near the Fermi level (*E_F_*), which is caused by the interfacial SOC that induces spin-split electronic states. This spin splitting is consistent with previous reports on SOC-induced band splitting in vdW heterostructure, indicating enhanced spin-dependent interaction, which is crucial for stabilizing DMI and Néel-type skyrmions [58,59].

To directly verify the critical role of spin–orbit coupling (SOC) in the interfacial DMI, we performed comparative first-principles calculations with and without SOC. In the absence of SOC, the heterostructure’s band structure remains spin-degenerate and unsplit near the Fermi level (Supplementary Figure S16a), and its total density of states (DOS) is entirely spin-symmetric (Supplementary Figure S16b). In contrast, with SOC included, the bands exhibit significant asymmetry and spin splitting (Figure 4d), and the DOS develops strong spin polarization near the Fermi level (Figure 4e), confirming that SOC is the central driver of the interfacial spin polarization.

Projected density of states (PDOS) analysis further reveals the specific contributions of atomic orbitals (Supplementary Figure S17). Under the influence of SOC, the Fe-3d orbitals exhibit strong spin-down polarization below the Fermi level, while the nearby Te-5p orbitals show spin-up polarization. These polarized states couple with the spin-polarized peaks of the Mo-4d orbitals from the MoS_2_ layer, forming a cross-interface spin-coupling chain. Without SOC, this chain is broken: only weak exchange splitting remains in the Fe-3d orbitals, while the Te-5p and Mo-4d orbitals show no spin polarization. This indicates that SOC is essential for establishing the interfacial spin-polarization transfer pathway. Distinct from the SOC-dependent polarization pathway shown in Supplementary Figure S17, a further PDOS comparison between the heterostructure and bulk Fe_3_GeTe_2_ (Supplementary Figure S18) highlights the significant enhancement of spin polarization in the Fe and Te orbitals at the interface. These results collectively demonstrate that the SOC-induced, orbitally selective spin polarization localized at the Fe/Te–Mo interface is the key microscopic mechanism responsible for generating the substantial DMI.

The density of states (DOS) provides additional evidence of interfacial spin polarization (Figure 4e). Pristine Fe_3_GeTe_2_ exhibits nearly symmetric spin-up and spin-down DOS, reflecting its centrosymmetric and non–spin-polarized electronic structure. In contrast, the heterostructure structure exhibits a strongly asymmetric DOS near *E_F_*, where the spin-up component is significantly enhanced relative to the spin-down component. This spin polarization is located at the Fe_3_GeTe_2_/MoS_2_ interface, confirming that proximity effects from MoS_2_ drive spin-dependent electronic reconstruction in Fe_3_GeTe_2_. Such asymmetric DOS is a key signature of interfacial DMI, because it reflects a preferred spin orientation required to stabilize the Néel-type skyrmion topology.

These results converge within a SOC-mediated interfacial coupling model (Figure 4f): The strong SOC of MoS_2_ induces spin polarization at the interface, which perturbs the adjacent Fe_3_GeTe_2_ magnetic moments, breaks inversion symmetry, and generates a finite DMI. The magnitude of this effect decays as the Fe_3_GeTe_2_ thickness increases, vanishing above ~60 nm, in agreement with the thickness-dependent skyrmion density observed experimentally. In summary, our findings establish interface engineering as a general route to stabilizing skyrmions in centrosymmetric van der Waals magnets. By correlating electronic reconstruction, SOC-driven symmetry breaking, and micromagnetic dynamics, we provide a mechanistic framework for designing tunable, low-power two-dimensional spintronic platforms.

## 4. Conclusions

In summary, we demonstrate that interfacial symmetry breaking in Fe_3_GeTe_2_/MoS_2_ heterostructures provides a universal mechanism for stabilizing Néel skyrmions in centrosymmetric vdW ferromagnets. While pristine Fe_3_GeTe_2_ exhibits no topological spin textures under zero-field cooling, the heterostructures host robust skyrmion lattices, directly evidencing the role of proximity-induced DMI. Thickness-dependent studies reveal maximal skyrmion density in thin Fe_3_GeTe_2_ layers (≤30 nm) and exponentially suppressed beyond ~60 nm, where bulk behavior recovers. Field-cooling protocols further enable zero-field metastability for spintronic applications. Both micromagnetic simulations and first-principles calculations confirm that interfacial DMI tilts moments toward chiral textures, with asymmetric charge redistribution and spin-split bands identified as the electronic origin. These results establish interface engineering as a deterministic strategy for controlling topological magnetism in vdW materials, opening a tunable platform for low-power racetrack memories and logic devices. Future investigations of current-driven skyrmion motion and its interaction with engineered defects in this system will be essential to assess its full technological potential.

## Data Availability

The original contributions presented in this study are included in the article/Supplementary Material. Further inquiries can be directed to the corresponding authors.

## Supplementary Materials

The following supporting information can be downloaded at: https://www.mdpi.com/article/10.3390/ma19020340/s1, Figure S1: Convergence tests for DFT calculations. (a) Total energy of MoS_2_ as a function of k-point sampling. (b) Total energy of Fe_3_GeTe_2_ as a function of k-point sampling. (c) Total energy of MoS_2_ as a function of plane-wave cutoff energy. (d) Total energy of Fe_3_GeTe_2_ as a function of plane-wave cutoff energy; Figure S2: Curie temperature of Fe_3_GeTe_2_. Temperature-dependent magnetization (M-T) curve measured with an out-of-plane magnetic field of 1000 Oe, indicating a Curie temperature (T_C_) of ~220 K; Figure S3: Perpendicular magnetic anisotropy of Fe_3_GeTe_2_ at cryogenic temperatures. Field-dependent magnetization (M-H) loops measured at 2 K −240 K with the magnetic field applied both out-of-plane and in-plane. The square hysteresis and higher remanence in the out-of-plane configuration confirm the magnetic easy axis is perpendicular to the sample plane, consistent with the out-of-plane magnetization of the skyrmions observed by LTEM; Figure S4: Magnetic force microscopy (MFM) image of the Fe_3_GeTe_2_/MoS_2_ heterostructure on SiN substrate. The red dashed line outlines the Fe_3_GeTe_2_ region, the yellow dashed line marks the MoS_2_ layer, and the blue dashed line indicates the SiN substrate (applied magnetic field: 317 Oe; scale bar: 2 μm); Figure S5: LTEM contrast simulation for a Néel-type skyrmion at various tilt angles. Schematic of the Néel-type spin configuration (from Refs. [48,49]) (a) and the corresponding simulated LTEM phase contrast under different sample tilts: (b) +11°, (c) 0°. The simulations demonstrate that observable magnetic phase contrast for this out-of-plane magnetized texture emerges only under tilted conditions, while it vanishes at normal incidence (b, 0° tilt). This result directly corroborates the experimental observations in Figure 2 and confirms the out-of-plane magnetization of the stabilized skyrmions; Figure S6: Magnetic domain observations in 30 nm Fe_3_GeTe_2_/4 nm MoS_2_ heterostructures under zero-field cooling (ZFC). Lorentz TEM images (11° tilt) showing Néel-type skyrmions (yellow circles) at varying magnetic fields (0–800 Oe). The dashed yellow line denotes the heterostructure boundary. Scale bar: 2 μm; Figure S7: Magnetic domain observations in 60 nm Fe_3_GeTe_2_/4 nm MoS_2_ heterostructures under zero-field cooling(ZFC). Lorentz TEM images (11° tilt) showing Néel-type skyrmions (yellow circles) at varying magnetic fields (0–800 Oe). The dashed yellow line denotes the heterostructure boundary. Scale bar: 2 μm; Figure S8: Statistical analysis of skyrmion nucleation and annihilation fields across multiple regions and independently fabricated heterostructures. (a–c) Representative Lorentz TEM images from three different regions of independently fabricated Fe_3_GeTe_2_ (45 nm)/MoS_2_ (4 nm) heterostructures, showing the magnetic field at which skyrmions first appear (nucleation) or completely vanish (annihilation); Figure S9: Statistical distribution of nucleation and annihilation fields. Histograms showing the distribution of (a) nucleation fields and (b) annihilation fields measured across three independent regions of the 30 nm, 45 nm and 60 nm Fe_3_GeTe_2_/4 nm MoS_2_ heterostructure under zero-field cooling conditions; Figure S10: Magnetic domain observations of 45 nm Fe_3_GeTe_2_/4 nm MoS_2_ heterostructures under field cooling (FC). Lorentz transmission electron microscopy images (tilted at 11°) show Néel-type skyrmions (yellow circles) under different magnetic fields (0–590 Oe). The yellow dotted line indicates the boundary of the heterostructure. Scale: 2 μm; Figure S11: Magnetic domain observations of 60 nm Fe_3_GeTe_2_/4 nm MoS_2_ heterostructures under field cooling (FC). Lorentz transmission electron microscopy images (tilted at 11°) show Néel-type skyrmions (yellow circles) under different magnetic fields (0–590 Oe). The yellow dotted line indicates the boundary of the heterostructure. Scale: 2 μm; Figure S12: Lorentz TEM characterization of a Fe_3_GeTe_2_ (30 nm)/MoS_2_ (~8 nm) heterostructure under zero-field cooling. The lower skyrmion density in the thicker MoS_2_ sample indicates that beyond an optimal thickness, additional MoS_2_ layers do not enhance the interfacial DMI efficiency; Figure S13: Size statistics of magnetic domain walls and Lorentz transmission electron microscope images in a 30 nm Fe_3_GeTe_2_/MoS_2_ heterostructure without applying a magnetic field during zero-field cooling (ZFC); Figure S14: The charge density and charge density difference in Fe_3_GeTe_2_; Figure S15: Spin-polarized band structure of Fe_3_GeTe_2_. The left and right panels correspond to spin-up and spin-down states, respectively; Figure S16: First-principles calculations without spin–orbit coupling (SOC). (a) Band structure of the Fe_3_GeTe_2_/MoS_2_ heterostructure calculated without SOC, showing spin-degenerate bands along the high-symmetry k-path. (b) Corresponding total density of states (TDOS) without SOC, illustrating the complete spin symmetry between spin-up and spin-down channels; Figure S17: Orbital-resolved projected density of states (PDOS) with and without SOC. PDOS for Fe-3d, Te-5p, and Mo-4d orbitals at the Fe_3_GeTe_2_/MoS_2_ interface. Solid and dashed lines represent spin-up and spin-down contributions, respectively. (a) Show results without SOC; (b) show results with SOC included. The SOC-induced spin polarization and interfacial coupling among Fe-3d, Te-5p, and Mo-4d orbitals are clearly visible; Figure S18: Comparative PDOS between the heterostructure and bulk Fe_3_GeTe_2_. PDOS of Fe-3d, Te-5p, and Mo-4d orbitals in the bulk Fe_3_GeTe_2_ (a) compared to those in SOC-included Fe_3_GeTe_2_/MoS_2_ heterostructure (b). The interfacial enhancement of spin polarization in Fe and Te orbitals, along with the emergence of Mo-4d spin polarization in the heterostructure, highlights the role of interfacial proximity and symmetry breaking. Note: The heterostructure data in (b) is shown for direct comparison with bulk properties, to highlight interface-induced enhancements in spin polarization and orbital coupling.

## References

[1] Wang Z., Zhang T., Ding M., Dong B., Li Y., Chen M., Li X., Huang J., Wang H., Zhao X. (2018). Electric-field control of magnetism in a few-layered van der Waals ferromagnetic semiconductor. Nat. Nanotechnol..

[2] Sun Y.P., Xiao R.C., Lin G.T., Zhang R.R., Ling L.S., Ma Z.W., Luo X., Lu W.J., Sheng Z.G. (2018). Effects of hydrostatic pressure on spin-lattice coupling in two-dimensional ferromagnetic Cr_2_Ge_2_Te_6_. Appl. Phys. Lett..

[3] Wang Z., Gutiérrez-Lezama I., Ubrig N., Kroner M., Gibertini M., Taniguchi T., Watanabe K., Imamoğlu A., Giannini E., Morpurgo A.F. (2018). Very large tunneling magnetoresistance in layered magnetic semiconductor CrI_3_. Nat. Commun..

[4] Li Q., Yang M., Gong C., Chopdekar R.V., N’Diaye A.T., Turner J., Chen G., Scholl A., Shafer P., Arenholz E. (2018). Patterning-Induced Ferromagnetism of Fe_3_GeTe_2_ van der Waals Materials beyond Room Temperature. Nano Lett..

[5] Klein D.R., MacNeill D., Song Q., Larson D.T., Fang S., Xu M., Ribeiro R.A., Canfield P.C., Kaxiras E., Comin R. (2019). Enhancement of interlayer exchange in an ultrathin two-dimensional magnet. Nat. Phys..

[6] Khela M., Da̧browski M., Khan S., Keatley P.S., Verzhbitskiy I., Eda G., Hicken R.J., Kurebayashi H., Santos E.J.G. (2023). Laser-induced topological spin switching in a 2D van der Waals magnet. Nat. Commun..

[7] Xu C., Feng J., Prokhorenko S., Nahas Y., Xiang H., Bellaiche L. (2020). Topological spin texture in Janus monolayers of the chromium trihalides Cr(I, X)3. Phys. Rev. B.

[8] Zhang X., Zhou Y., Song K.M., Park T.-E., Xia J., Ezawa M., Liu X., Zhao W., Zhao G.-P., Woo S. (2020). Skyrmion-electronics: Writing, deleting, reading and processing magnetic skyrmions toward spintronic applications. J. Phys. Condens. Matter.

[9] Liu M., Wan T.L., Dou K., Zhang L., Sun W., Jiang J., Ma Y., Gu Y., Kou L. (2024). Magnetic skyrmions and their manipulations in a 2D multiferroic CuCrP_2_Te_6_ monolayer. Phys. Chem. Chem. Phys..

[10] Kong L., Tang J., Wu Y., Wang W., Jiang J., Wang Y., Li J., Xiong Y., Wang S., Tian M. (2024). Diverse helicities of dipolar skyrmions. Phys. Rev. B.

[11] Cheng S., Bagués N., Selcu C.M., Freyermuth J.B., Li Z., Wang B., Das S., Hammel P.C., Randeria M., McComb D.W. (2023). Room-temperature magnetic skyrmions in Pt/Co/Cu multilayers. Phys. Rev. B.

[12] Nagaosa N., Tokura Y. (2013). Topological properties and dynamics of magnetic skyrmions. Nat. Nanotechnol..

[13] Niu H., Yoon H.G., Kwon H.Y., Cheng Z., Fu S., Zhu H., Miao B., Sun L., Wu Y., Schmid A.K. (2025). Magnetic skyrmionic structures with variable topological charges in engineered Dzyaloshinskii-Moriya interaction systems. Nat. Commun..

[14] Cheng T.C., Zhang L., Kurokawa Y., Satone R., Tokunaga K., Yuasa H. (2025). Computational study of skyrmion stability and transport on W/CoFeB. Sci. Rep..

[15] Ma M., Pan Z., Ma F. (2022). Artificial skyrmion in magnetic multilayers. J. Appl. Phys..

[16] Sampaio J., Cros V., Rohart S., Thiaville A., Fert A. (2013). Nucleation, stability and current-induced motion of isolated magnetic skyrmions in nanostructures. Nat. Nanotechnol..

[17] Dzyaloshinsky I. (1958). A thermodynamic theory of “weak” ferromagnetism of antiferromagnet-ics. J. Phys. Chem. Solids.

[18] Moriya T. (1960). Anisotropic Superexchange Interaction and Weak Ferromagnetism. Phys. Rev..

[19] Muhlbauer S., Binz B., Jonietz F., Pfleiderer C., Rosch A., Neubauer A., Georgii R., Boni P. (2009). Skyrmion Lattice in a Chiral Magnet. Science.

[20] Yu X.Z., Onose Y., Kanazawa N., Park J.H., Han J.H., Matsui Y., Nagaosa N., Tokura Y. (2010). Real-space observation of a two-dimensional skyrmion crystal. Nature.

[21] Li Y., Basnet R., Pandey K., Hu J., Wang W., Ma X., McCray A.R.C., Petford-Long A.K., Phatak C. (2022). Field-Dependent Magnetic Domain Behavior in van der Waals Fe_3_GeTe_2_. JOM.

[22] Tran H.B., Matsushita Y.-I. (2024). Skyrmions in van der Waals centrosymmetric materials with Dzyaloshinskii–Moriya interactions. Scr. Mater..

[23] May A.F., Calder S., Cruz C.D., McGuire M.A., Yan J.-Q., Sales B.C. (2016). Magnetic struc-ture and phase stability of the van der Waals bonded ferromagnet Fe_3_-xGeTe_2_. Phys. Rev. B.

[24] Deng Y., Yu Y., Song Y., Zhang J., Wang N.Z., Sun Z., Yi Y., Wu Y.Z., Wu S., Zhu J. (2018). Gate-tunable room-temperature ferromagnetism in two-dimensional Fe_3_GeTe_2_. Nature.

[25] Wu Y., Zhang S., Zhang J., Wang W., Zhu Y.L., Hu J., Yin G., Wong K., Fang C., Wan C. (2020). Néel-type skyrmion in WTe_2_/Fe_3_GeTe_2_ van der Waals heterostructure. Nat. Commun..

[26] Ganguly S., Neelam, Grinberg I., Margel S. (2021). Layer by layer controlled synthesis at room temperature of tri-modal (MRI, fluorescence and CT) core/shell superparamagnetic IO/human serum albumin nanoparticles for diagnostic applications. Polym. Adv. Technol..

[27] Rahmani A.A., Jia Q., Bahti H.H., Fauzia R.P., Wyantuti S. (2025). Recent advances in lanthanide-based nanoparticle contrast agents for magnetic resonance imaging: Synthesis, characterization, and applications. OpenNano.

[28] Tsai L.-Z., Jain R.K., Lin Y.-T., Hai N.T., Wu C.-C., Liang J.-Z., Lee Y.-H., Lee S.-F. (2025). Interfacial DMI in stamp-transferred monolayer-WS_2_/Py heterostructure. J. Magn. Magn. Mater..

[29] Fedel S., Villa M., Damerio S., Demiroglu E., Deger C., Gazquez J., Avci C.O. (2025). Evidence of Long-Range Dzyaloshinskii–Moriya Interaction at Ferrimagnetic Insulator/Nonmagnetic Metal Interfaces. Adv. Funct. Mater..

[30] Samardak A.S., Ognev A.V., Kolesnikov A.G., Stebliy M.E., Samardak V.Y., Iliushin I.G., Pervishko A.A., Yudin D., Platunov M., Ono T. (2022). XMCD and ab initio study of interface-engineered ultrathin Ru/Co/W/Ru films with perpendicular magnetic anisotropy and strong Dzyaloshinskii–Moriya interaction. Phys. Chem. Chem. Phys..

[31] Zhu L., Zhu L., Ma X., Li X., Buhrman R.A. (2022). Critical role of orbital hybridization in the Dzyaloshinskii-Moriya interaction of magnetic interfaces. Commun. Phys..

[32] Yun J., Cui B., Cui Q., He X., Chang Y., Zhu Y., Yan Z., Guo X., Xie H., Zhang J. (2023). Anisotropic Interlayer Dzyaloshinskii–Moriya Interaction in Synthetic Ferromagnetic/Antiferromagnetic Sandwiches. Adv. Funct. Mater..

[33] Luo Y.K., Xu J., Zhu T., Wu G., McCormick E.J., Zhan W., Neupane M.R., Kawakami R.K. (2017). Opto-Valleytronic Spin Injection in Monolayer MoS_2_/Few-Layer Graphene Hybrid Spin Valves. Nano Lett..

[34] Wakamura T., Reale F., Palczynski P., Guéron S., Mattevi C., Bouchiat H. (2018). Strong Aniso-tropic Spin-Orbit Interaction Induced in Graphene by Monolayer WS_2_. Phys. Rev. Lett..

[35] Wang Z., Ki D.-K., Khoo J.Y., Mauro D., Berger H., Levitov L.S., Morpurgo A.F. (2016). Origin and Magnitude of ‘Designer’ Spin-Orbit Interaction in Graphene on Semiconducting Transition Metal Dichalcogenides. Phys. Rev. X.

[36] Yang B., Tu M.-F., Kim J., Wu Y., Wang H., Alicea J., Wu R., Bockrath M., Shi J. (2016). Tunable spin–orbit coupling and symmetry-protected edge states in graphene/WS_2_. 2D Mater..

[37] Avsar A., Tan J.Y., Taychatanapat T., Balakrishnan J., Koon G.K.W., Yeo Y., Lahiri J., Carvalho A., Rodin A.S., O’Farrell E.C.T. (2014). Spin–orbit proximity effect in graphene. Nat. Commun..

[38] Zhong D., Seyler K.L., Linpeng X., Cheng R., Sivadas N., Huang B., Schmidgall E., Taniguchi T., Watanabe K., McGuire M.A. (2017). Van der Waals engineering of ferromagnetic semiconductor heterostructures for spin and valleytronics. Sci. Adv..

[39] Seyler K.L., Zhong D., Huang B., Linpeng X., Wilson N.P., Taniguchi T., Watanabe K., Yao W., Xiao D., McGuire M.A. (2018). Valley Manipulation by Optically Tuning the Magnetic Proximity Effect in WSe_2_/CrI_3_ Heterostructures. Nano Lett..

[40] Zhang Z., Ni X., Huang H., Hu L., Liu F. (2019). Valley splitting in the van der Waals hetero-structure Wse_2_/CrI_3_: The role of atom superposition. Phys. Rev. B.

[41] Xie J., Jia L., Shi H., Yang D., Si M. (2019). Electric field mediated large valley splitting in the van der Waals heterostructure WSe_2_/CrI_3_. Jpn. J. Appl. Phys..

[42] Lu B., Ali A., Muhammad I., Zhou S., Zhang W. (2025). Ferromagnetic Fe_3_GeTe_2_/semiconducting SiSb van der Waals spin-interface. Surf. Interfaces.

[43] Peng L., Yasin F.S., Park T.-E., Kim S.J., Zhang X., Nagai T., Kimoto K., Woo S., Yu X. (2021). Tunable Néel–Bloch Magnetic Twists in Fe_3_GeTe_2_ with van der Waals Structure. Adv. Funct. Mater..

[44] Park T.-E., Peng L., Liang J., Hallal A., Yasin F.S., Zhang X., Song K.M., Kim S.J., Kim K., Weigand M. (2021). Néel-type skyr-mions and their current-induced motion in van der Waals ferromagnet-based hetero-structures. Phys. Rev. B.

[45] Powalla L., Birch M.T., Litzius K., Wintz S., Schulz F., Weigand M., Scholz T., Lotsch B.V., Kern K., Schütz G. (2022). Single Skyrmion Generation via a Vertical Nanocontact in a 2D Magnet-Based Heterostructure. Nano Lett..

[46] Weerahennedige H., Irziqat M., Vithanage D., Weerarathne H., Ronau Z., Sumanasekera G., Jasinski J.B. (2024). The effects of thickness, polarization, and strain on vibrational modes of 2D Fe_3_GeTe_2_. Surf. Interfaces.

[47] Cortijo-Campos S., Kung P., Prieto C., de Andrés A. (2021). Forbidden and Second-Order Phonons in Raman Spectra of Single and Few-Layer MoS_2_ Close to C Exciton Resonance. J. Phys. Chem. C.

[48] Fert A., Cros V., Sampaio J. (2013). Skyrmions on the track. Nat. Nanotechnol..

[49] Lv X., Lv H., Huang Y., Zhang R., Qin G., Dong Y., Liu M., Pei K., Cao G., Zhang J. (2024). Distinct skyrmion phases at room temperature in two-dimensional ferromagnet Fe_3_GaTe_2_. Nat. Commun..

[50] Tolley R., Montoya S.A., Fullerton E.E. (2018). Room-temperature observation and current control of skyrmions in Pt/Co/Os/Pt thin films. Phys. Rev. Mater..

[51] Yu G., Jenkins A., Ma X., Razavi S.A., He C., Yin G., Shao Q., He Q.L., Wu H., Li W. (2018). Room-Temperature Skyrmions in an Antiferromagnet-Based Heterostructure. Nano Lett..

[52] Mandru A.-O., Yıldırım O., Tomasello R., Heistracher P., Penedo M., Giordano A., Suess D., Finocchio G., Hug H.J. (2020). Coexistence of distinct skyrmion phases observed in hybrid ferromagnetic/ferrimagnetic multilayers. Nat. Commun..

[53] May A.F., Ovchinnikov D., Zheng Q., Hermann R., Calder S., Huang B., Fei Z., Liu Y., Xu X., McGuire M.A. (2019). Ferromagnetism Near Room Temperature in the Cleavable van der Waals Crystal Fe_3_GeTe_2_. ACS Nano.

[54] Meng L., Wang Z., Zhang Y., Cai S., Yin Z., Chen R., Zhao Y., Peng Y., Shi Y., Li S. (2021). Anomalous thickness dependence of Curie temperature in air-stable two-dimensional ferromagnetic 1T-CrTe_2_ grown by chemical vapor deposition. Nat. Commun..

[55] León-Brito N., Bauer E.D., Ronning F., Thompson J.D., Movshovich R. (2016). Magnetic microstructure and magnetic properties of uniaxial itinerant ferromagnet Fe_3_GeTe_2_. J. Appl. Phys..

[56] Kim D., Park S., Lee J., Yoon J., Joo S., Kim T., Min K.-J., Park S.-Y., Kim C., Moon K.-W. (2019). Antiferromagnetic coupling of van der Waals ferromagnetic Fe_3_GeTe_2_. Nanotechnology.

[57] Vansteenkiste A., Leliaert J., Dvornik M., Helsen M., Garcia-Sanchez F., Van Waeyen-berge B. (2014). The design and verification of MuMax3. AIP Adv..

[58] You B., Wang X., Mi W. (2015). Prediction of spin–orbital coupling effects on the electronic structure of two dimensional van der Waals heterostructures. Phys. Chem. Chem. Phys..

[59] Li X.-H., Wang B.-J., Cai X.-L., Zhang L.-W., Wang G.-D., Ke S.-H. (2017). Tunable electronic properties of arsenene/GaS van der Waals heterostructures. RSC Adv..

