# Supplementary material for: Creation of High-Density Néel Skyrmions by Interfacial-Proximity Engineering
Source: Materials (Basel). 2026 Jan 14;19(2):340. doi: 10.3390/ma19020340 (PMC12843417; doi:10.3390/ma19020340)
Supplement: Supplementary file 1 [file materials-19-00340-s001.zip › materials-4086226-supplementary.pdf]

Supplementary Information

Creation of High-Density Néel Skyrmions by Interfacial-Proximity Engineering

Tingjia Zhang [1,2], Chendi Yang [2], Xiaowei Lv [2], Ke Pei [3], Xiao Yang [2], Wuyang Tan [2], Junye Pan [4], Jiazhuan Qin [2], Meichen Wen [5], Wei Li [1,*], Jia Liang [6,*] and Renchao Che [2,*]

[1] School of Materials and Chemistry, University of Shanghai for Science and Technology, Shanghai 200093, China; 211550185@st.usst.edu.cn
[2] Laboratory of Advanced Materials, Shanghai Key Lab of Molecular Catalysis and Innovative Materials, State Key Laboratory of Coatings for Advanced Equipment, College of Smart Materials and Future Energy, Fudan University, Shanghai 200438, China; 20113010017@fudan.edu.cn (C.Y.); 21113010017@m.fudan.edu.cn (X.L.); 24213010014@m.fudan.edu.cn (X.Y.); 24213010012@m.fudan.edu.cn (W.T.); 24110300029@m.fudan.edu.cn (J.Q.)
[3] Electron Microscopy Center, Institute of Science and Technology, Fudan University, Shanghai 200438, China; 18113010016@fudan.edu.cn
[4] College of Physics, Donghua University, Shanghai 201620, China; 1259229@mail.dhu.edu.cn
[5] Materials Genome Institute, Shanghai University, Shanghai 200444, China; meichen1022@foxmail.com
[6] College of Smart Materials and Future Energy, State Key Laboratory of Photovoltaic Science and Technology, Fudan University, Shanghai 200433, China
* Correspondence: liwei176@usst.edu.cn (W.L.); jialiang@fudan.edu.cn (J.L.); rcche@fudan.edu.cn (R.C.)

Note on Supplementary Figures

The Supplementary Information provides a comprehensive dataset that extends and underpins the concise findings presented in the main text. The figures are complementary, not duplicative:

Supplementary Figure S1 presents the convergence tests for density functional theory (DFT) calculations.

Supplementary Figures S2–3 provide the basic magnetic properties of Fe_3GeTe_2, including its Curie temperature and perpendicular magnetic anisotropy, which serve as a foundation for understanding the observed skyrmion behavior.

Supplementary Figure S4 shows the LTEM images of the Fe_3GeTe_2/MoS_2 heterostructure on a silicon nitride (SiN) substrate.

Supplementary Figure S5 shows the simulated LTEM images of a Neel-type domain wall at 0° and tilted sample angles.

Supplementary Figures S6–9 present magnetic domain observations and statistical analysis for 30 nm and 60 nm Fe_3GeTe_2/MoS_2 heterostructures under zero-field cooling (ZFC) conditions, along with the statistical distribution of nucleation and annihilation fields across different Fe_3GeTe_2 thicknesses. These form a key basis for the statistical analyses in the main text.

Supplementary Figures S10–11 present magnetic domain observations for 45 nm and 60 nm Fe_3GeTe_2/MoS_2 heterostructures under field cooling (FC) conditions, complementing the ZFC dataset.

Supplementary Figure S12 shows the Lorentz TEM characterization of a Fe_3GeTe_2 (30 nm)/MoS_2 (~8 nm) heterostructure under zero-field cooling, illustrating the effect of MoS_2 thickness on skyrmion density.

Supplementary Figure S13 provides the size statistics of magnetic domain walls and corresponding Lorentz TEM images in a 30 nm Fe_3GeTe_2/MoS_2 heterostructure under zero-field cooling (ZFC).

Supplementary Figures S14–15 offer detailed first-principles calculation results for the pristine Fe_3GeTe_2, specifically its charge density difference and spin-polarized band structure. These are presented as a reference and comparison to highlight the distinct electronic modifications induced at the heterointerface.

Supplementary Figures S16–18 provide additional detailed electronic structure analyses for the Fe_3GeTe_2/MoS_2 heterostructure and comparative data with pristine Fe_3GeTe_2, including calculations without spin–orbit coupling (SOC), orbital-resolved projected density of states (PDOS) with/without SOC, and comparative PDOS.

These results underpin the mechanistic discussion of interfacial proximity effects in the main text.

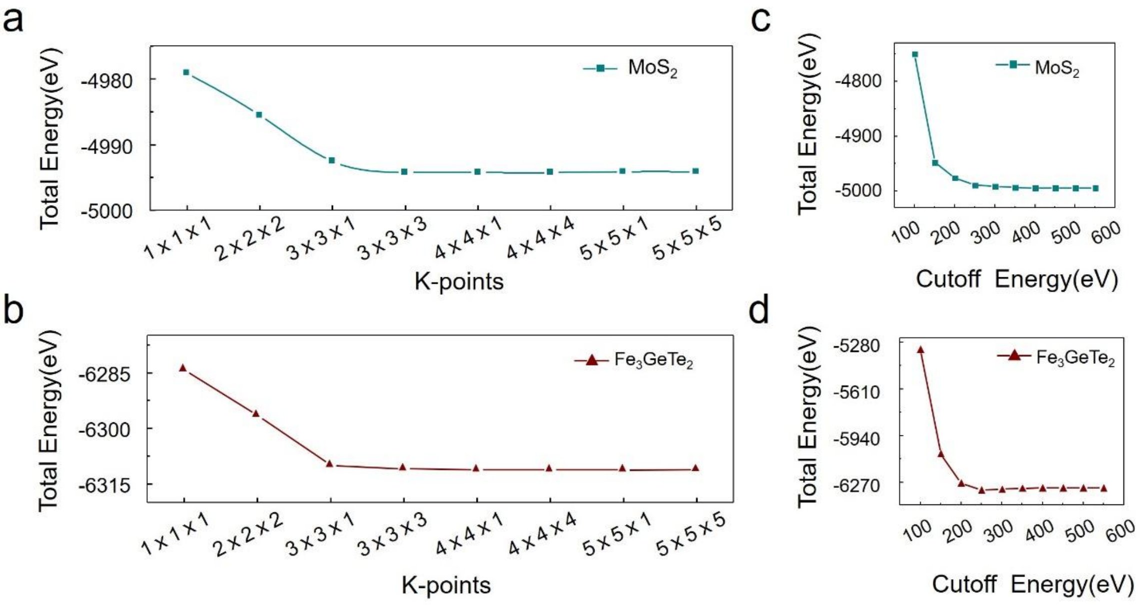

Supplementary Figure S1. Convergence tests for DFT calculations. (a) Total energy of MoS_2 as a function of k-point sampling. (b) Total energy of Fe_3GeTe_2 as a function of k-point sampling. (c) Total energy of MoS_2 as a function of plane-wave cutoff energy. (d) Total energy of Fe_3GeTe_2 as a function of plane-wave cutoff energy.

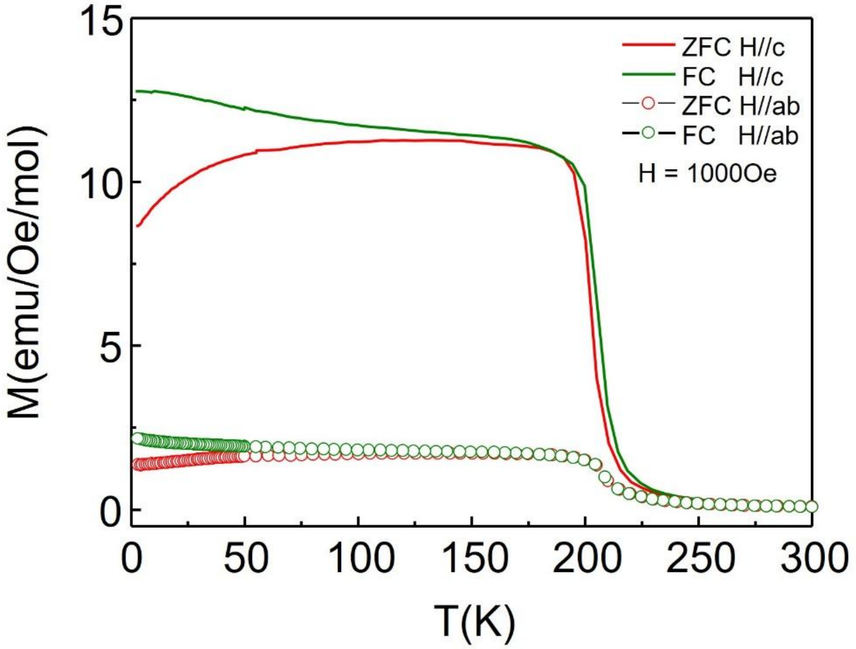

Supplementary Figure S2. Curie temperature of Fe_3GeTe_2. Temperature-dependent magnetization (M-T) curve measured with an out-of-plane magnetic field of 1000 Oe, indicating a Curie temperature (T_C) of ~220 K.

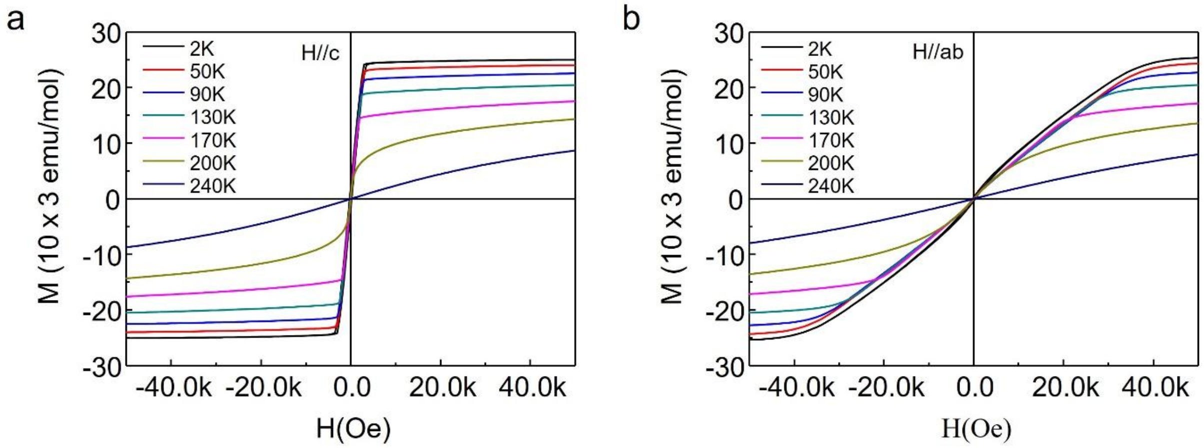

Supplementary Figure S3. Perpendicular magnetic anisotropy of Fe_3GeTe_2 at cryogenic temperatures. Field-dependent magnetization (M-H) loops measured at 2 K -240 K with the magnetic field applied both out-of-plane and in-plane. The square hysteresis and higher remanence in the out-of-plane configuration confirm the magnetic easy axis is perpendicular to the sample plane, consistent with the out-of-plane magnetization of the skyrmions observed by LTEM.

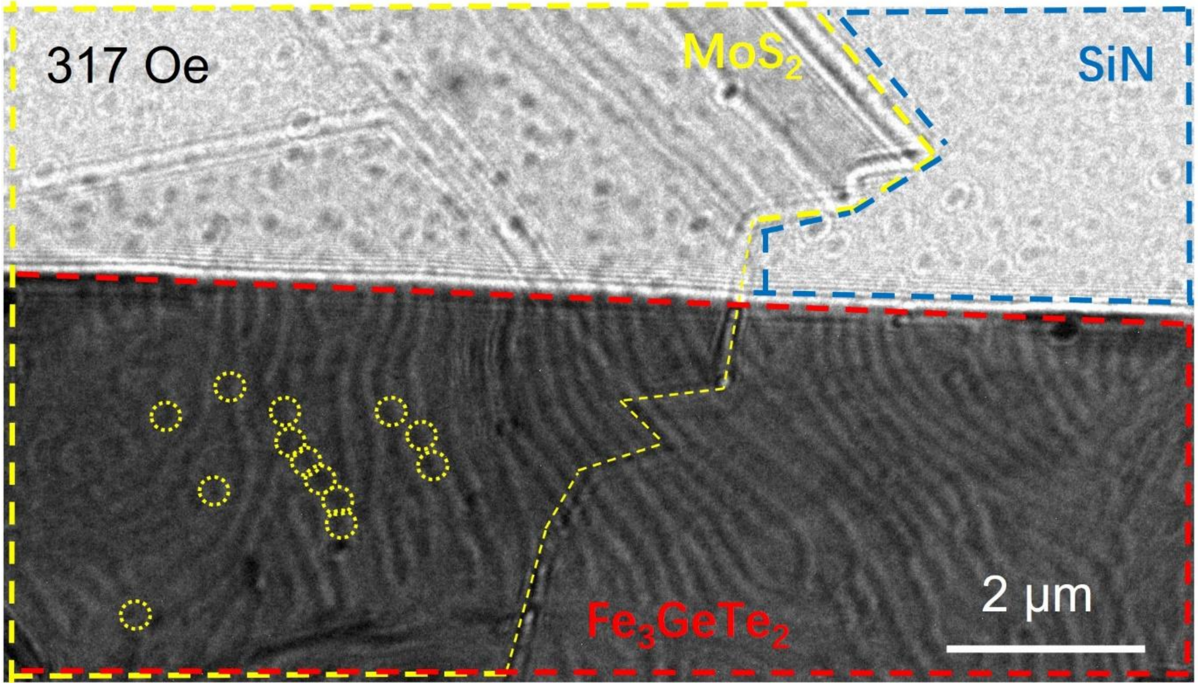

Supplementary Figure S4. Magnetic force microscopy (MFM) image of the Fe_3GeTe_2/MoS_2 heterostructure on SiN substrate. The red dashed line outlines the Fe_3GeTe_2 region, the yellow dashed line marks the MoS_2 layer, and the blue dashed line indicates the SiN substrate (applied magnetic field: 317 Oe; scale bar: 2 μm).

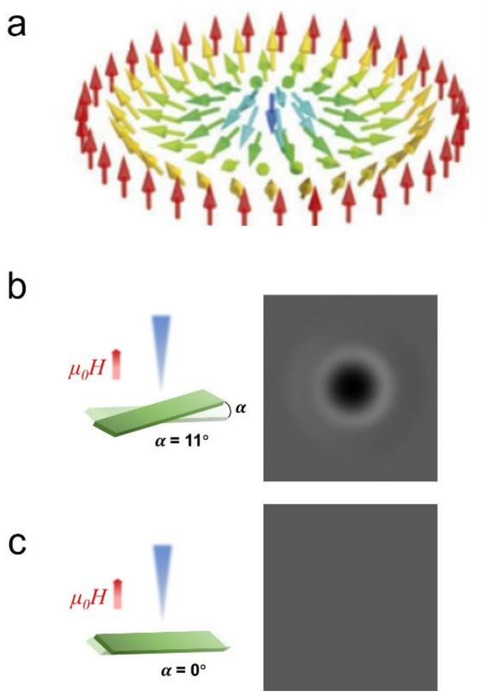

Supplementary Figure S5. LTEM contrast simulation for a Néel-type skyrmion at various tilt angles. Schematic of the Néel-type spin configuration (from Ref. [48, 49]) (a) and the corresponding simulated LTEM phase contrast under different sample tilts: (b) +11°, (c) 0°. The simulations demonstrate that observable magnetic phase contrast for this out-of-plane magnetized texture emerges only under tilted conditions, while it vanishes at normal incidence (b, 0° tilt). This result directly corroborates the experimental observations in Fig. 2 and confirms the out-of-plane magnetization of the stabilized skyrmions.

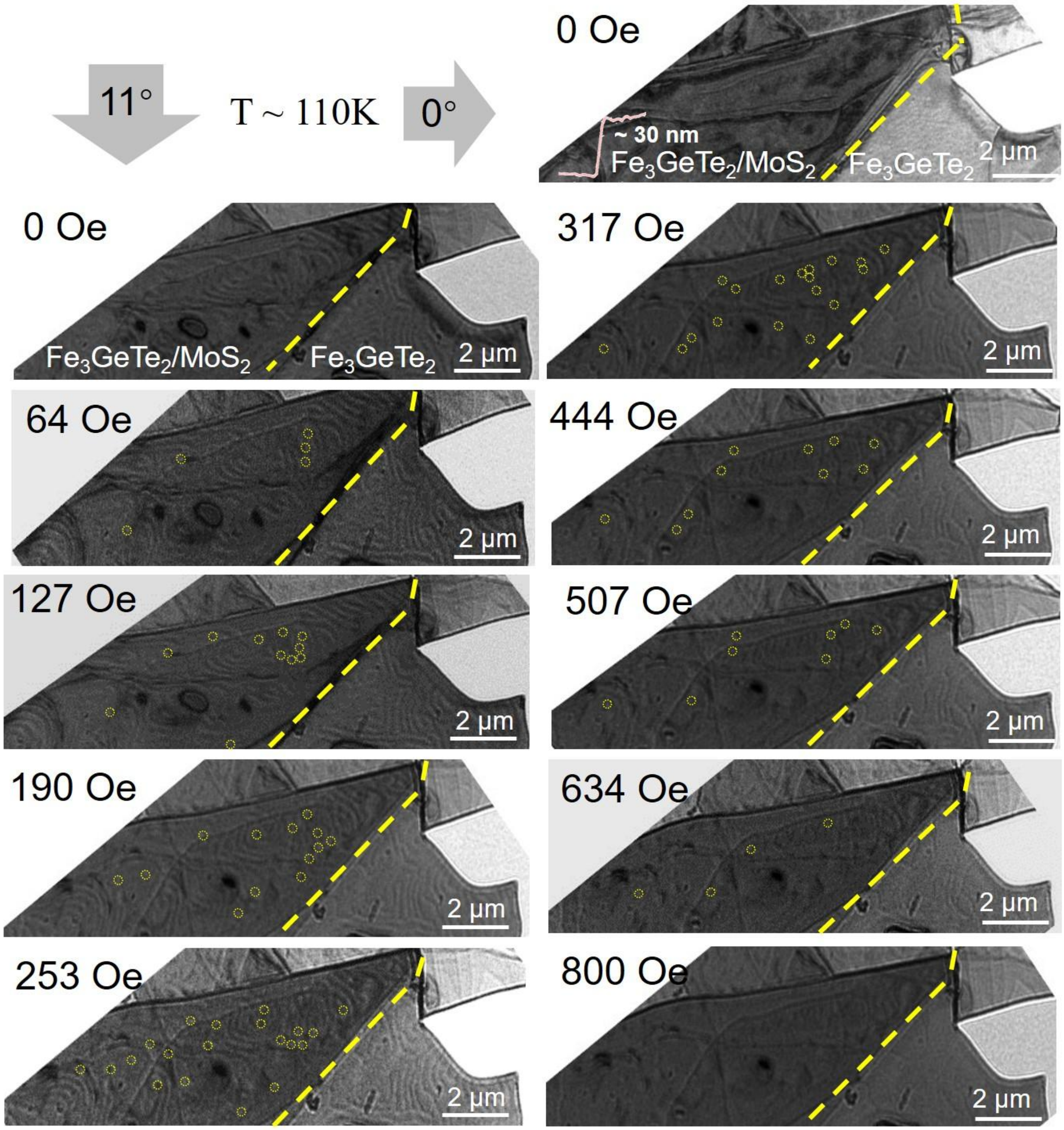

Supplementary Figure S6. Magnetic domain observations in 30nm Fe_3GeTe_2/4nm MoS_2 heterostructures under zero-field cooling(ZFC). Lorentz TEM images (11° tilt) showing Néel-type skyrmions (yellow circles) at varying magnetic fields (0–800 Oe). The dashed yellow line denotes the heterostructure boundary. Scale bar: 2 μm.

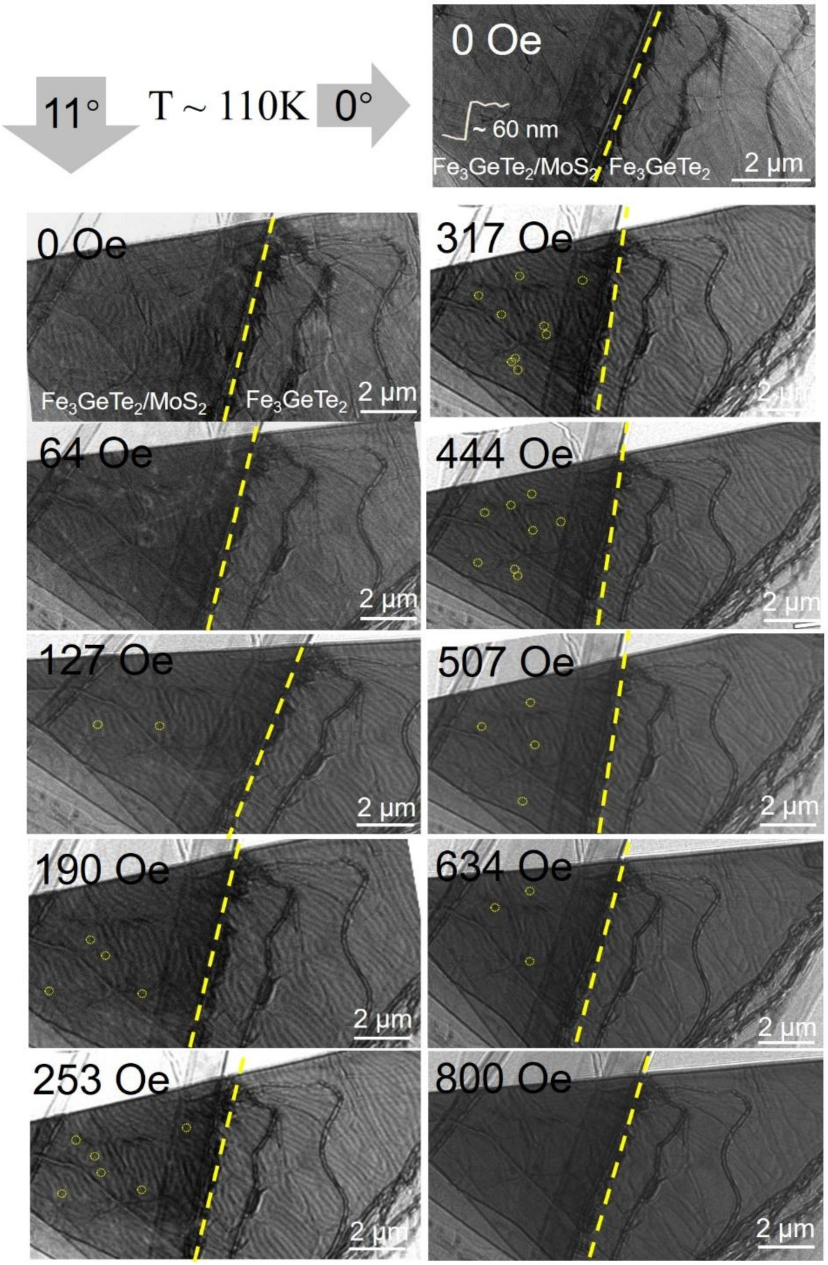

Supplementary Figure S7. Magnetic domain observations in 60nm Fe_3GeTe_2/4nm MoS_2 heterostructures under zero-field cooling(ZFC). Lorentz TEM images (11° tilt) showing Néel-type skyrmions (yellow circles) at varying magnetic fields (0–800 Oe). The dashed yellow line denotes the heterostructure boundary. Scale bar: 2 μm.

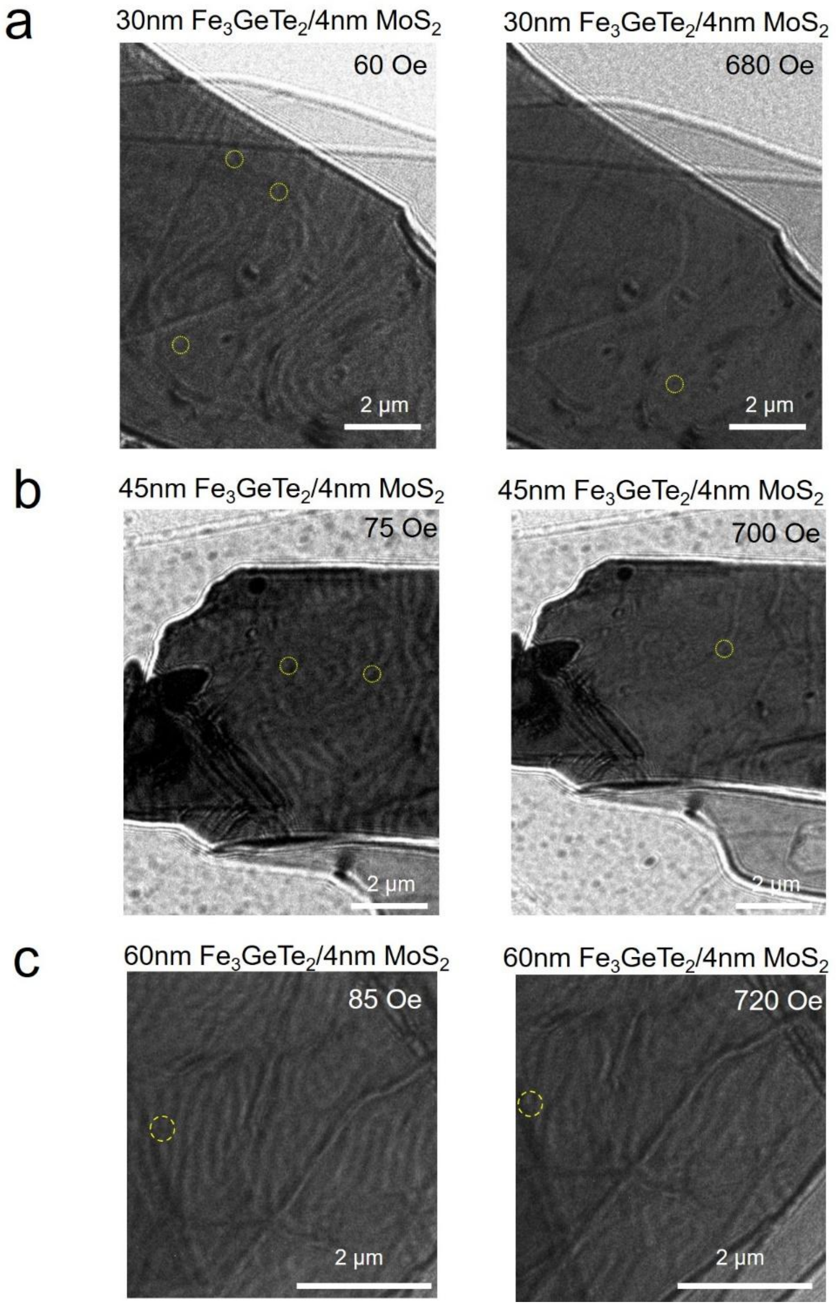

Supplementary Figure S8. **Statistical analysis of skyrmion nucleation and annihilation fields across multiple regions and independently fabricated heterostructures.** (a-c) Representative Lorentz TEM images from three different regions of independently fabricated Fe_3GeTe_2 (45 nm)/MoS_2 (4 nm) heterostructures, showing the magnetic field at which skyrmions first appear (nucleation) or completely vanish (annihilation).

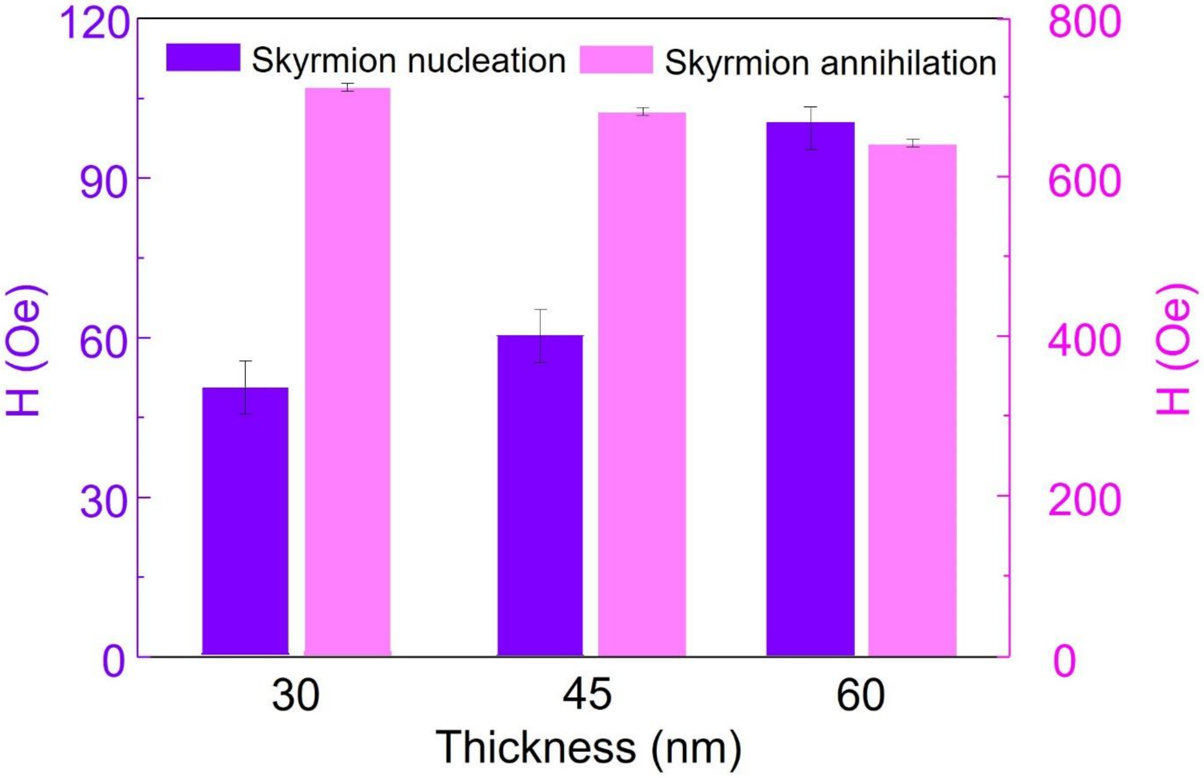

Supplementary Figure S9 Statistical distribution of nucleation and annihilation fields. Histograms showing the distribution of (a) nucleation fields and (b) annihilation fields measured across three independent regions of the 30 nm, 45 nm and 60nm Fe_3GeTe_2/4 nm MoS_2 heterostructure under zero-field cooling conditions.

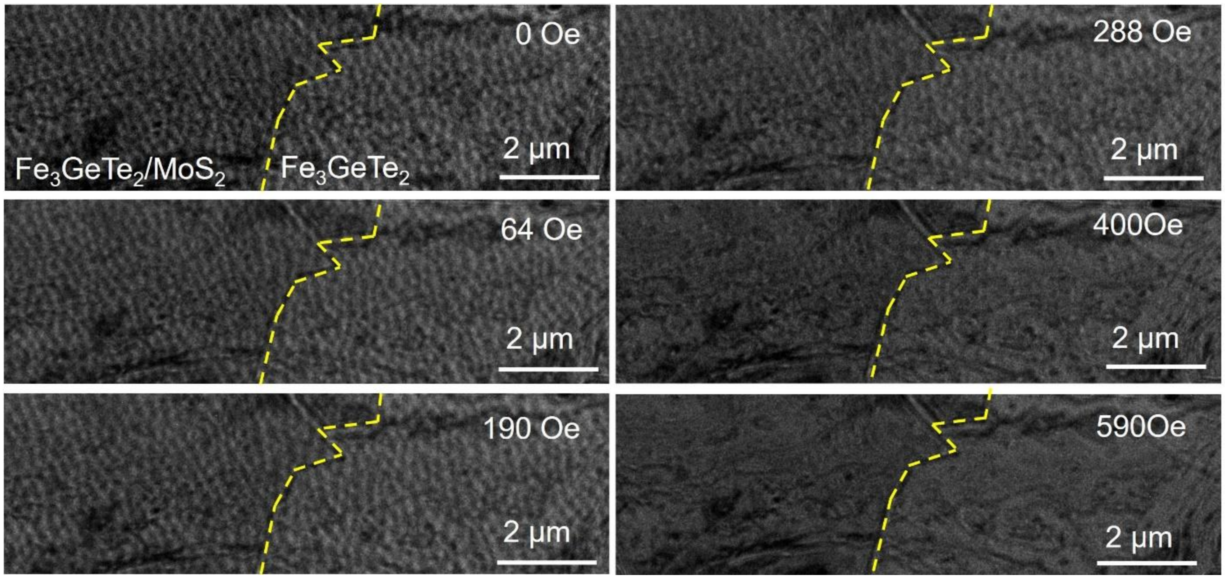

Supplementary Figure S10. Magnetic domain observations of 45 nm Fe_3GeTe_2/4 nm MoS_2 heterostructures under field cooling(FC). Lorentz transmission electron microscopy images (tilted at 11°) show Néel-type skyrmions (yellow circles) under different magnetic fields (0-590 Oe). The yellow dotted line indicates the boundary of the heterostructure. Scale: 2 μm.

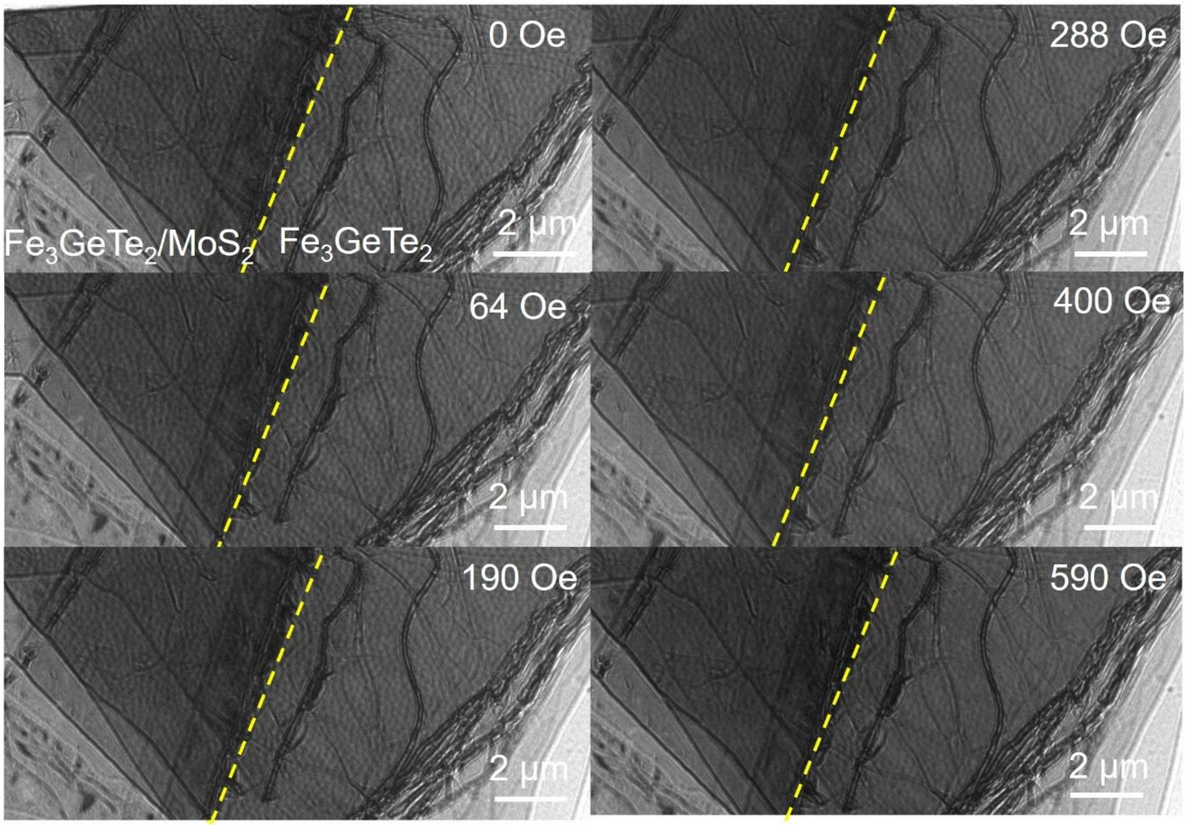

Supplementary Figure S11. Magnetic domain observations of 60 nm Fe_3GeTe_2/4 nm MoS_2 heterostructures under field cooling(FC). Lorentz transmission electron microscopy images (tilted at 11°) show Néel-type skyrmions (yellow circles) under different magnetic fields (0-590 Oe). The yellow dotted line indicates the boundary of the heterostructure. Scale: 2 μm.

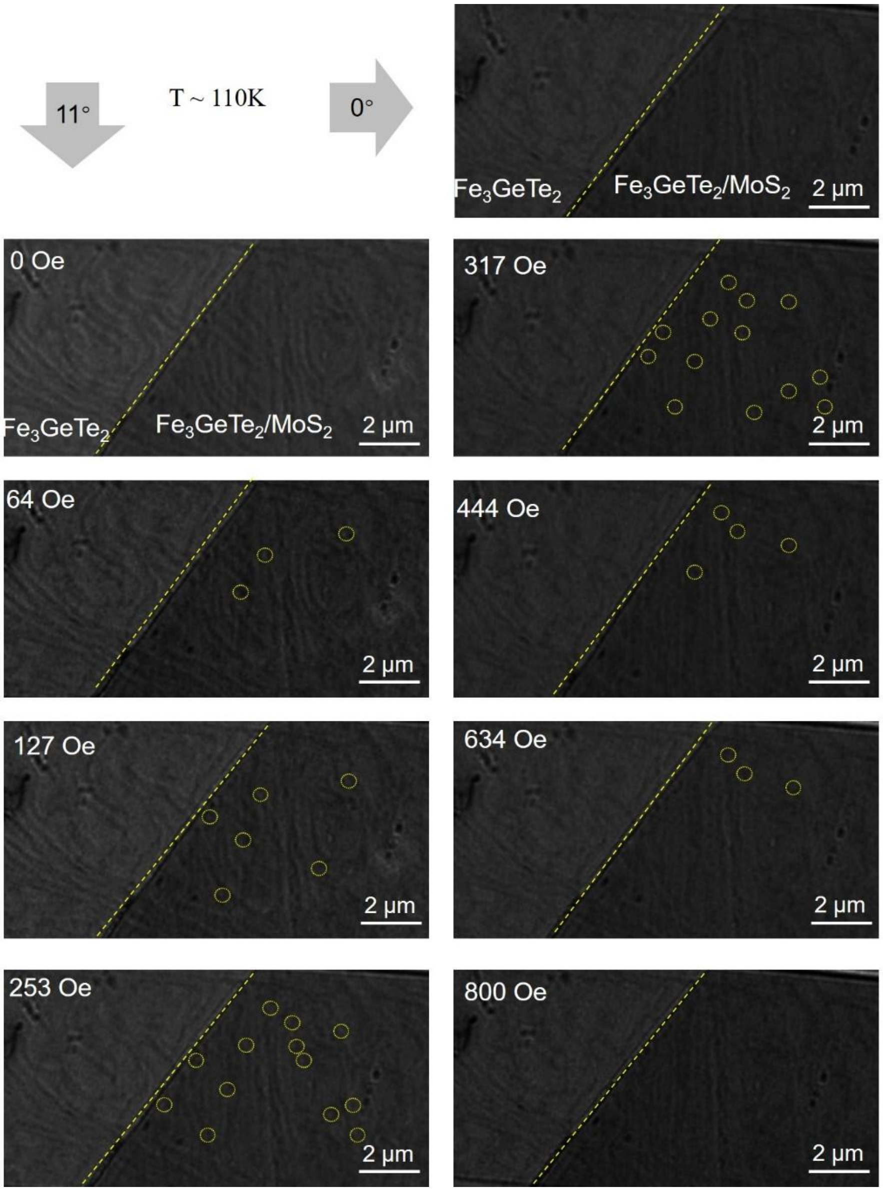

Supplementary Figure S12. Lorentz TEM characterization of a Fe_3GeTe_2 (30 nm)/MoS_2 (~8 nm) heterostructure under zero-field cooling. The lower skyrmion density in the thicker MoS_2 sample indicates that beyond an optimal thickness, additional MoS_2 layers do not enhance the interfacial DMI efficiency.

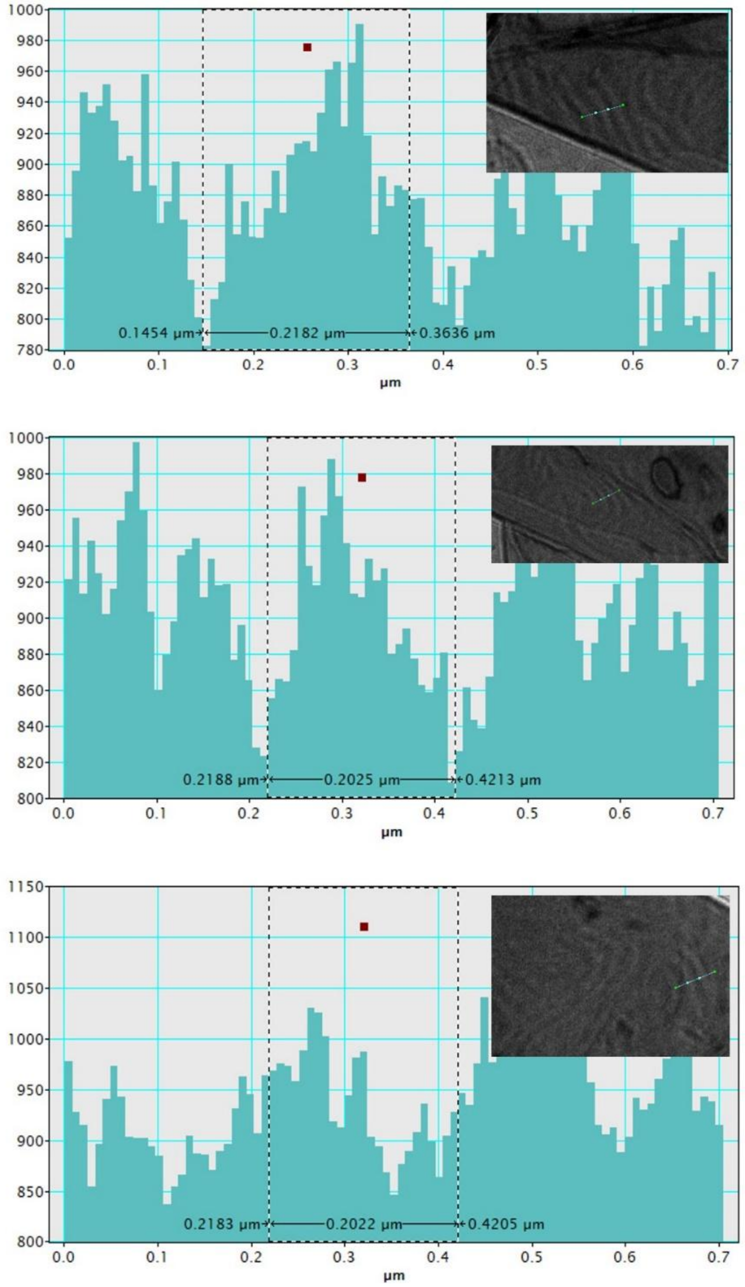

Supplementary Figure S13. Size statistics of magnetic domain walls and Lorentz transmission electron microscope images in a 30nm Fe_3GeTe_2/MoS_2 heterostructure without applying a magnetic field during zero-field cooling (ZFC).

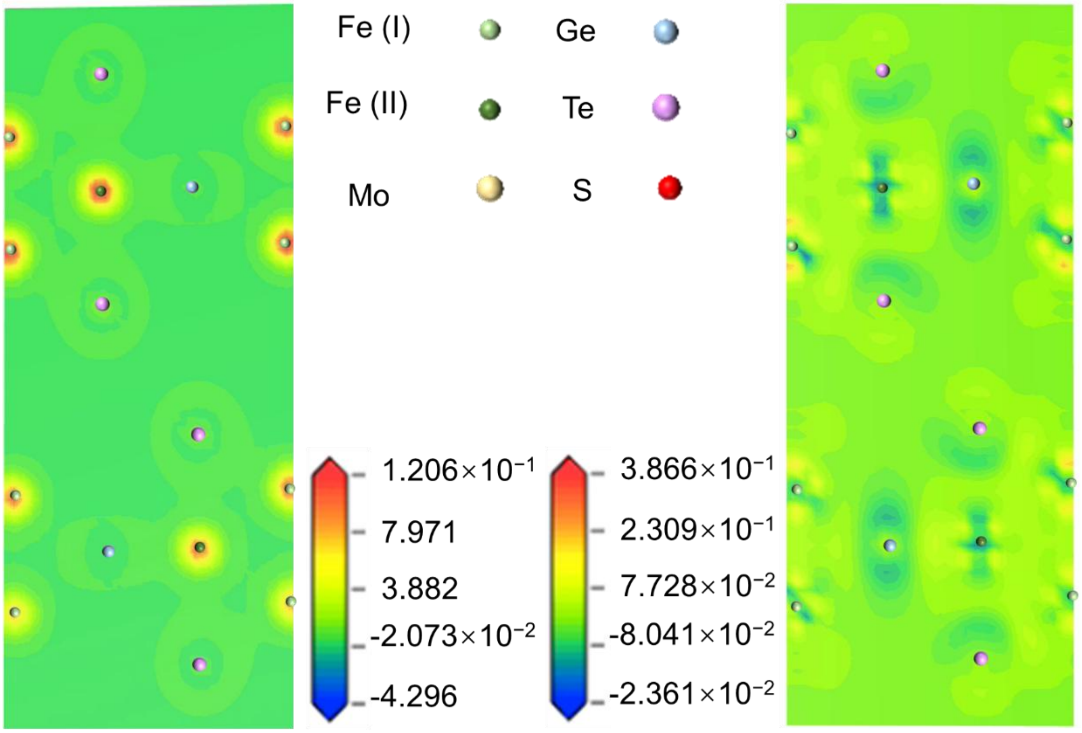

Supplementary Figure S14. The charge density and charge density difference in Fe_3GeTe_2.

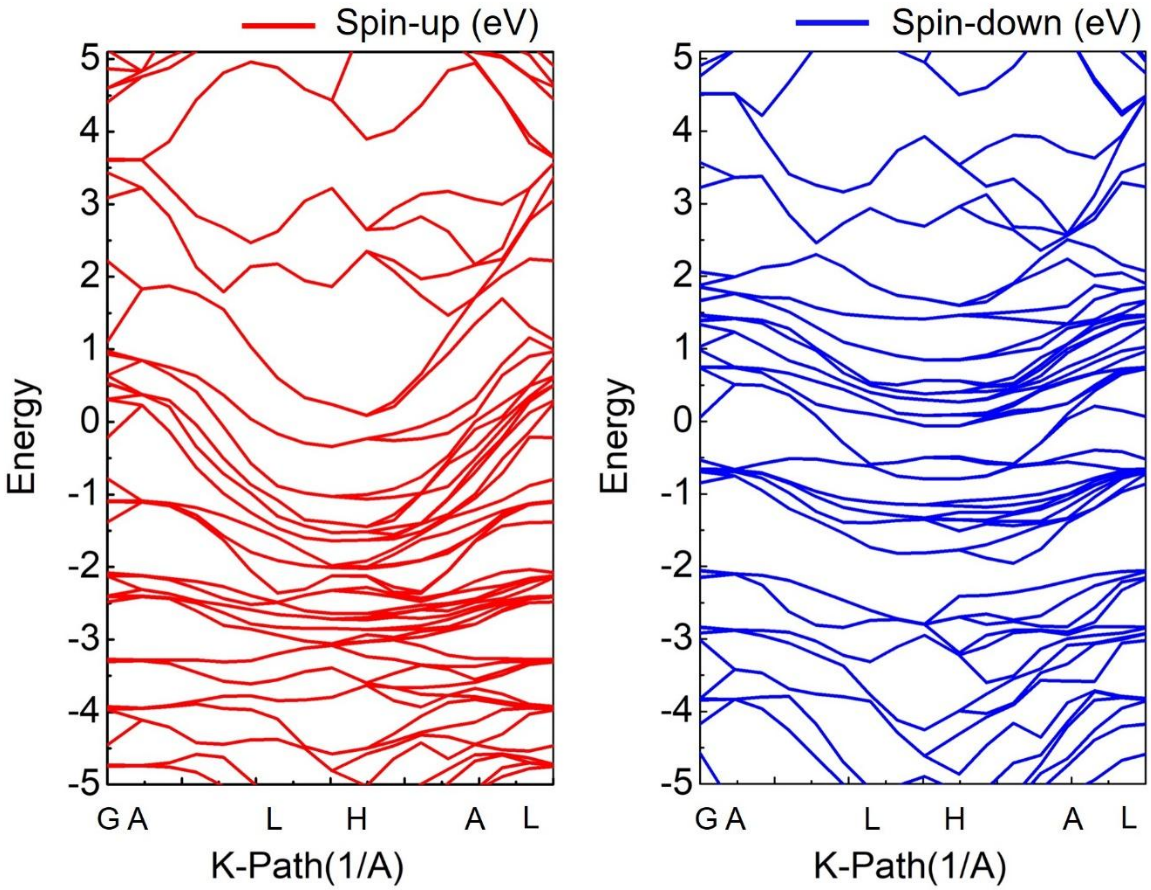

Supplementary Figure S15. Spin-polarized band structure of Fe_3GeTe_2. The left and right panels correspond to spin-up and spin-down states, respectively.

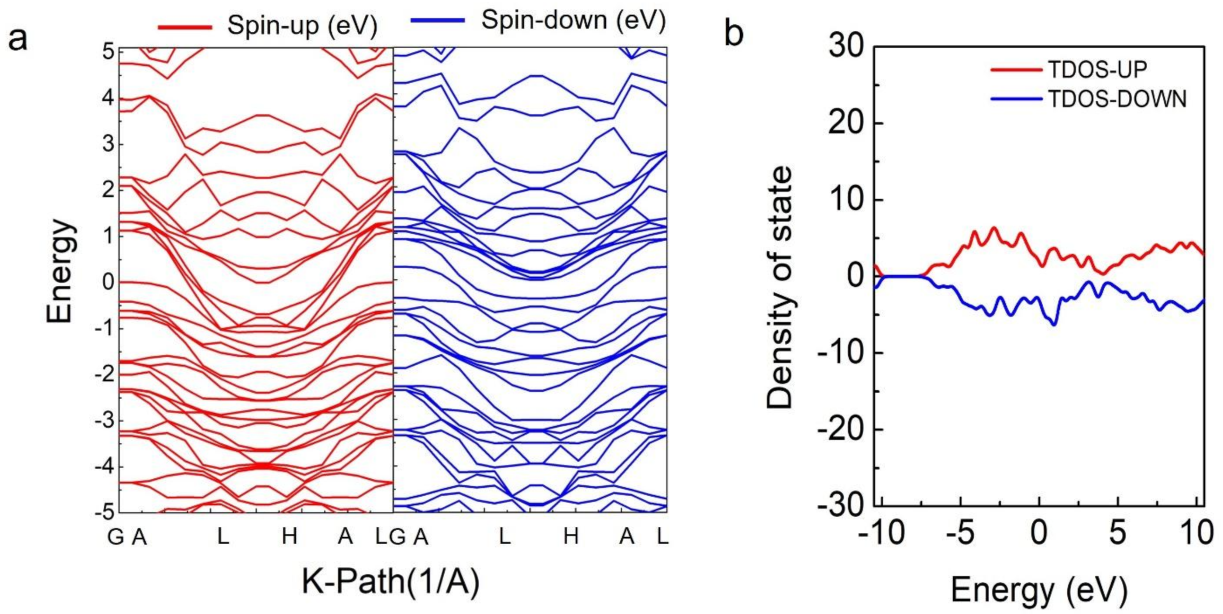

Supplementary Figure S16. First-principles calculations without spin–orbit coupling (SOC). (a) Band structure of the Fe_3GeTe_2/MoS_2 heterostructure calculated without SOC, showing spin-degenerate bands along the high-symmetry k-path. (b) Corresponding total density of states (TDOS) without SOC, illustrating the complete spin symmetry between spin-up and spin-down channels.

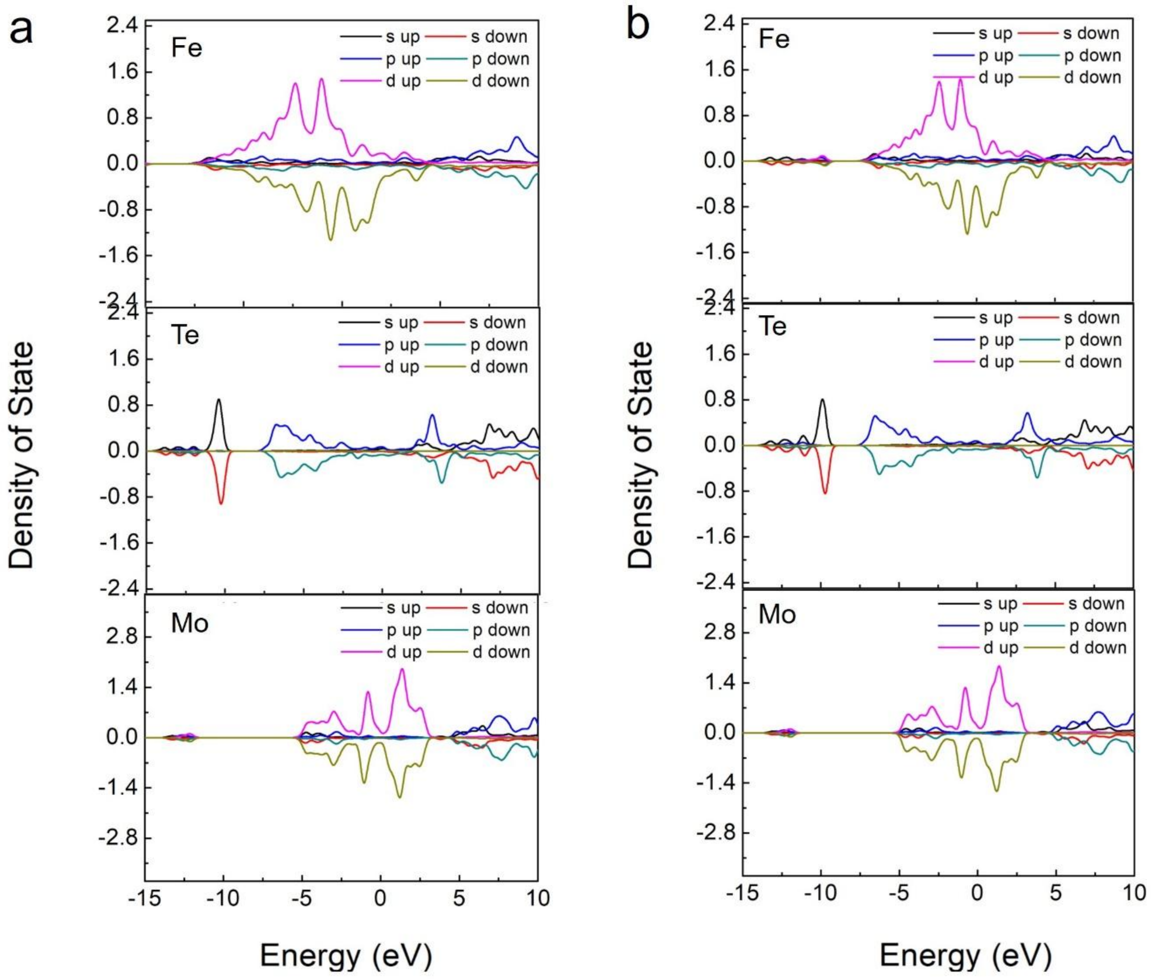

Supplementary Figure S17. Orbital-resolved projected density of states (PDOS) with and without SOC. PDOS for Fe-3d, Te-5p, and Mo-4d orbitals at the Fe_3GeTe_2/MoS_2 interface. Solid and dashed lines represent spin-up and spin-down contributions, respectively. (a) Show results without SOC; (b) show results with SOC included. The SOC-induced spin polarization and interfacial coupling among Fe-3d, Te-5p, and Mo-4d orbitals are clearly visible.

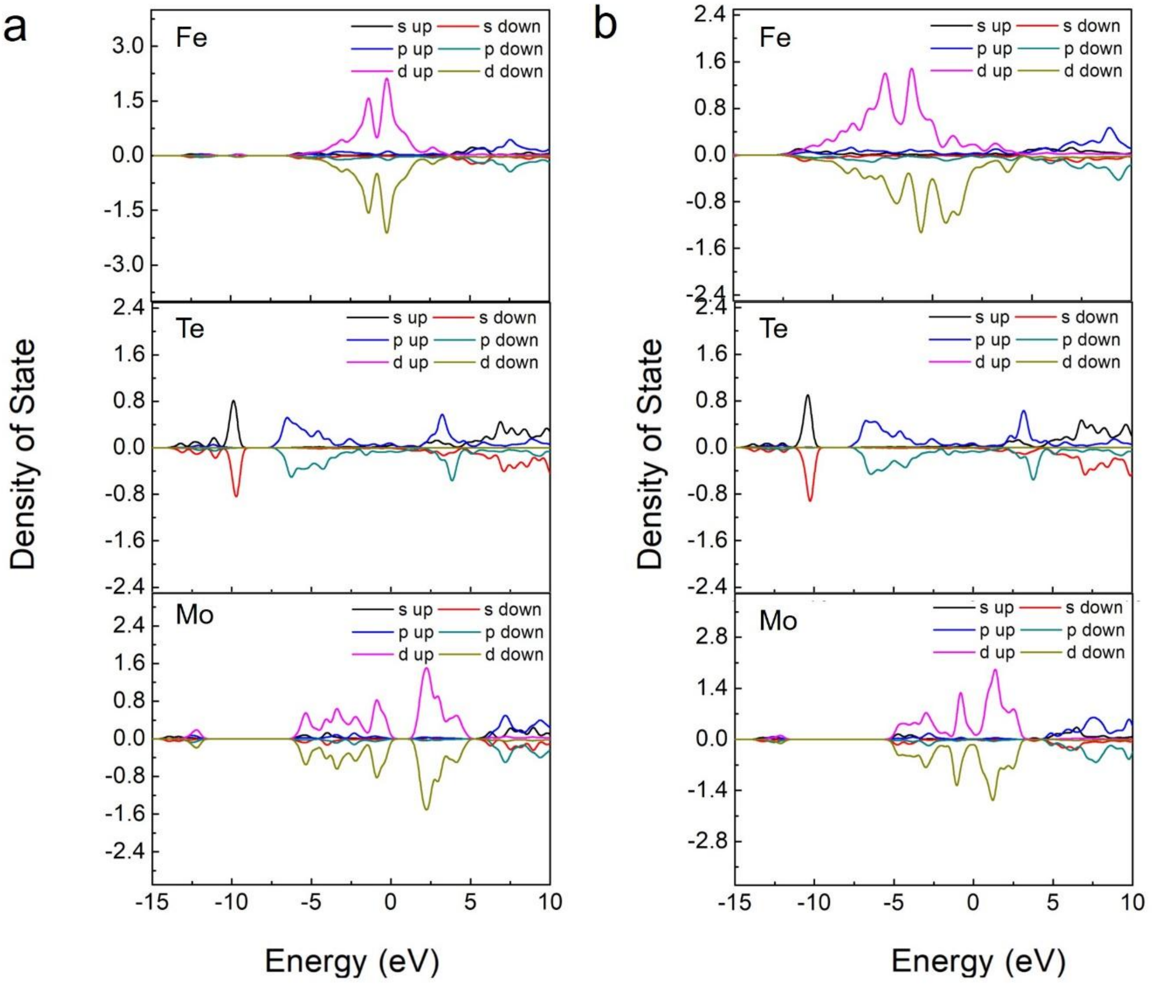

Supplementary Figure S18. Comparative PDOS between the heterostructure and bulk Fe_3GeTe_2. PDOS of Fe-3d, Te-5p, and Mo-4d orbitals in the bulk Fe_3GeTe_2 (a) compared to those in SOC-included Fe_3GeTe_2/MoS_2 heterostructure (b). The interfacial enhancement of spin polarization in Fe and Te orbitals, along with the emergence of Mo-4d spin polarization in the heterostructure, highlights the role of interfacial proximity and symmetry breaking. Note: The heterostructure data in (b) is shown for direct comparison with bulk properties, to highlight interface-induced enhancements in spin polarization and orbital coupling.
